# Spatio‐Temporal Diversity of Calcium Activity in Microglia

**DOI:** 10.1002/glia.70131

**Published:** 2026-01-06

**Authors:** Hiroshi Horiuchi, Dennis Lawrence Cheung, Junko Ishida, Junichi Nabekura

**Affiliations:** ^1^ Division of Homeostatic Development National Institute for Physiological Science, National Institutes of Natural Sciences Okazaki Japan; ^2^ Department of Physiological Sciences SOKENDAI: The Graduate University for Advanced Studies Hayama Japan; ^3^ Institute for Research on Next‐Generation Semiconductor and Sensing Science (IRES^2^), Toyohashi University of Technology Toyohashi Japan; ^4^ Department of Developmental and Regenerative Neurobiology Nagoya City University Graduate School of Medical Sciences Nagoya Japan

**Keywords:** awake state, Ca^2+^ imaging, in vivo two‐photon imaging, microglia, neuronal activity, P2Y12, purinergic signaling

## Abstract

Microglia, the brain's innate immune cells, possess complex, highly motile branched processes. These act independently, enabling individual processes to carry out entirely distinct functions in parallel. Intracellular Ca^2+^ signaling is implicated in many of these distinct microglial functions. However, it has been difficult to quantify how such Ca^2+^ activity is compartmentalized in space and time to prevent unwanted cross‐talk between signaling pathways. Previous studies have typically relied on manually drawn regions‐of‐interest (ROIs), which averages fluorescence within predefined compartments and therefore cannot resolve the fine‐scale spatio‐temporal propagation patterns that may be functionally relevant. To address this, we adopt an unbiased non‐ROI‐based analytical approach to comprehensively characterize the temporal, spatial and spatio‐temporal dimensions of microglial Ca^2+^ activity in vivo. We find that microglial Ca^2+^ activity predominantly occurs in processes, tends to remain localized at its site of origin, and, when it propagates, often follows a well‐defined direction (either toward or away from the soma) rather than spreading isotropically as would be expected under purely passive diffusion. The tendency of microglial Ca^2+^ activity to spread between intracellular regions does not correlate with peak amplitude, but appears to be limited by the branching points of the microglial processes. Finally, we show that Ca^2+^ activity can differ between the microglial soma and its processes in response to various pharmacological stimuli. These results suggest that Ca^2+^ signals are actively compartmentalized within microglia in a context dependent manner, rather than being synchronized across the entire cell.

## Graphical abstract

Ca^2+^ activity mostly occurs in microglial processes and stays localized. When it spreads, it often shows a directional bias and is constrained by branch points. Activity strongly relies on P2Y12 receptors and is shaped by neuronal activity.

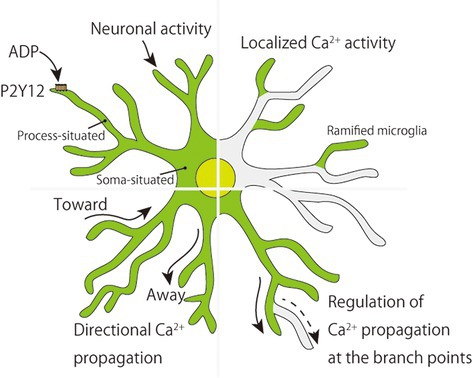

## 1 Introduction

Microglia are the sole resident immune cells in the central nervous system (CNS). However, the diverse functions they perform extend beyond this label (Kettenmann et al. 2011). Under pathological conditions, microglia adopt an amoeboid morphology and neutralize pathogens and debris through phagocytosis. Under physiological conditions, microglia maintain a ramified morphology and continuously surveil the surrounding extracellular milieu by extending and retracting their processes (Davalos et al. 2005; Nimmerjahn et al. 2005). As part of this surveillance, microglia directly contact neighboring neuronal somas, axons, dendrites, and spines with their ramified processes (Cserep et al. 2020; Li et al. 2012; Tremblay et al. 2010; Wake et al. 2009). This activity, which is greatly influenced by neuronal activity itself, exerts diverse effects on the structure and function of neuronal circuits (Dissing‐Olesen et al. 2014; Eyo et al. 2014). In different contexts, physiological microglia–neuron contact may promote filopodia formation on dendrites (Miyamoto et al. 2016), enhance synaptic transmission (Akiyoshi et al. 2018), or facilitate removal of spines and even whole neurons (Li et al. 2012).

Notably, microglial processes appear to operate independently of each other, with no overall synchronicity or coordination in their motility patterns (Davalos et al. 2005; Nimmerjahn et al. 2005). A single microglia can simultaneously have some processes actively engaged in phagocytosis, while other processes remain uninvolved, maintaining a ramified morphology (Kamei and Okabe 2023; Luo et al. 2016; Sierra et al. 2010). On the other hand, widespread changes in the surrounding environment, such as injury or increased neuronal circuit activity, can induce synchronized motility between processes (Davalos et al. 2005; Eyo et al. 2014; Haynes et al. 2006). Taken together, this suggests that the behavior of each microglial process is governed by stimuli within its immediate surroundings.

These characteristics of microglial process motility point to upstream regulatory intracellular signaling cascades that are both amenable to modulation by extracellular stimuli and subject to strict subcellular compartmentalization. Thus, spatial and temporal characterization of these intracellular signaling cascades, which are presently poorly understood, may be informative for decoding microglial functions. For this purpose, Ca^2+^ imaging is a promising approach given the importance of Ca^2+^ as a second messenger in intracellular signaling cascades.

A number of previous studies have examined the Ca^2+^ activity of in vitro cultured microglia (Ferrari et al. 1996; Hoffmann et al. 2003; Korvers et al. 2016; Langfelder et al. 2015; Moller et al. 1997, 2000; Walz et al. 1993), ex vivo microglia in acutely prepared brain slices (Moller et al. 2000), and in vivo microglia in healthy and diseased brains (Brawek et al. 2017; Eichhoff et al. 2011; Liu et al. 2021; Pozner et al. 2015; Umpierre et al. 2020). However, their differing experimental conditions are an important consideration when comparing results, as the microglial phenotype is exquisitely sensitive to environmental factors. Nevertheless, most studies agree that microglial Ca^2+^ activity is minimal under physiological conditions (Brawek et al. 2017; Eichhoff et al. 2011; Pozner et al. 2015; Umpierre et al. 2020), influenced by extracellular and intracellular factors (Langfelder et al. 2015; Moller et al. 2000), and increases significantly in pathological contexts (e.g., LPS‐induced immunological activation, laser‐induced brain injury, neural hyperactivity, epileptic seizures, and stroke) (Brawek et al. 2017; Eichhoff et al. 2011; Liu et al. 2021; Pozner et al. 2015; Umpierre et al. 2020). Interestingly, however, anesthesia can reduce or increase microglial Ca^2+^ activity depending on the precise context (Umpierre et al. 2020). Specific signaling molecules that stimulate microglial Ca^2+^ activity (e.g., ATP and complement factors) (Farber and Kettenmann 2006; Ferrari et al. 1996; Korvers et al. 2016; Moller 2002; Moller et al. 1997; Walz et al. 1993), and those released by microglia when intracellular levels Ca^2+^ are high (e.g., nitric oxide, cytokines (Hoffmann et al. 2003)) have also been identified.

However, previous studies have been more limited in quantifying the dynamics of microglial Ca^2+^ activity due to their reliance on region‐of‐interest (ROI)‐based analysis methods (Eichhoff et al. 2011; Pozner et al. 2015; Umpierre et al. 2020). Whilst temporal parameters (e.g., Ca^2+^ event amplitude, frequency, and duration) can be quantified using such methods, spatio‐temporal parameters (e.g., Ca^2+^ event onset location, diffusibility, direction, velocity) cannot. Addressing this gap is important as various patterns of microglial Ca^2+^ activity (e.g., Ca^2+^ waves) are speculated to serve important biological functions but have yet to be comprehensively quantified (Liu et al. 2021; Pozner et al. 2015; Umpierre et al. 2020).

Thus, in this study, we employed the non‐ROI‐based AQuA (Wang et al. 2019) image analysis platform to extract Ca^2+^ events from in vivo two‐photon Ca^2+^ imaging data of microglia in healthy brains of awake mice. We first confirm that AQuA is able to extract basic temporal dynamics parameters about microglial Ca^2+^ activity (i.e., event frequency, amplitude, and duration), which have been partially characterized by ROI‐based analysis approaches in previous studies. We next use AQuA to extract more sophisticated spatial and spatio‐temporal dynamics parameters about microglial Ca^2+^ activity (i.e., event onset location, propagation propensity, directionality, and speed), which have not been previously described. Finally, we perform pharmacological investigations into the underlying mechanisms of the observed microglial Ca^2+^ activity.

## 2 Results

The signaling mechanisms that enable microglia to control their individual processes independently are not known. To address this, we imaged microglial Ca^2+^ activity in vivo and analyzed its spatio‐temporal dynamics using an unbiased non‐ROI‐based pipeline.

### 2.1 Basic Characterization of Microglial Ca^2+^ Activity Dynamics Using a Non‐ROI‐Based Approach

High quality in vivo imaging of microglial Ca^2+^ activity requires the presence of fluorescent probes within the intracellular milieu that are sensitive to Ca^2+^, bright, and have a high signal‐to‐noise ratio. To achieve this, we developed a new genetically modified mouse strain by crossing the Iba1‐tTA (Tanaka et al. 2012) and the tetO‐GCaMP6 (Ohkura et al. 2012) mouse strains. Thus, in the resulting Iba1‐GCaMP6 mouse strain, expression of the genetically encoded Ca^2+^ indicator GCaMP6 was coupled to the microglia‐specific promoter Iba1 via the tetracycline conditional (tTA transcription factor, tetO promoter region) expression system (Figure S1A). Using immunohistochemistry, we confirmed that this facilitated exclusive expression of GCaMP6 in microglia (Figure S1B).

**FIGURE 1:**
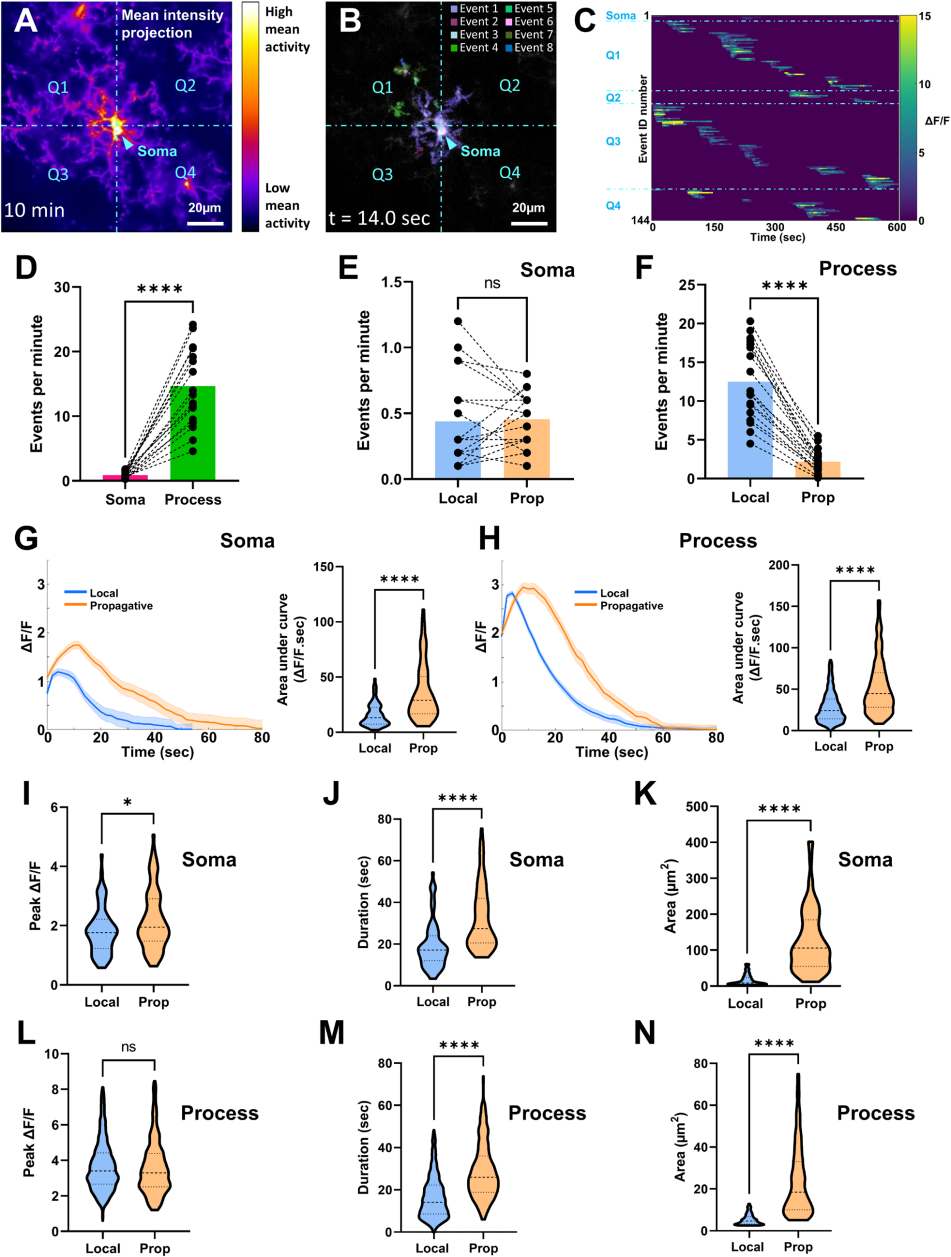
Characterization of microglial Ca^2+^ activity as discrete Ca^2+^ events. (A–C) AQuA‐based deconvolution of GCaMP6 activity fluorescence into discrete Ca^2+^ events in a representative microglia. The field of view is divided into four quadrants (Q1, Q2, Q3, and Q4) with the microglial soma at its center (arrowhead). Scale bar = 20 μm. (A) Microglial morphology is reconstructed from the mean intensity projection of GCaMP6 fluorescence across all image frames acquired over the 10‐min observation period. Thus, the color scale is indicative of the average level of Ca^2+^ activity exhibited within the various subcellular regions of the microglia during this time period. Note that this reconstruction approach flattens the microglia's morphology from three‐dimensional (five *Z*‐planes) to two‐dimensional. (B) During AQuA‐based deconvolution, extracted Ca^2+^ events are mapped to the reconstructed two‐dimensional morphological map of the microglia. In the displayed excerpt at *t* = 14 s, eight discrete Ca^2+^ events occur (color key). Note the variation in the locations and area sizes of these Ca^2+^ events. (C) Raster plot of all Ca^2+^ events occurring during the 10‐min observation period. Each of the 144 rows (left *Y*‐axis) represents a single discrete Ca^2+^event. The rows have been grouped based on the Ca^2+^ event location (Soma, Q1, Q2, Q3, and Q4). The duration (*X*‐axis) and evolution of activity intensity (color scale) of each Ca^2+^ event is indicated. (D–F) Dot plots quantifying the frequency of microglial Ca^2+^ events as classified by their location and spreading dynamics. Each pair of data points represents one microglia cell, *n* = 18. Columns indicate cohort means. ****p < 0.0001; Wilcoxon's matched‐pairs signed rank test, two‐tailed. (D) All Ca^2+^ events per microglia: Soma‐situated (Soma) versus process‐situated (Process). (E) Soma‐situated Ca^2+^ events: Localized (Local) versus propagative (Prop). (F) Process‐situated Ca^2+^ events: Localized (Local) versus propagative (Prop). (G, H) Microglial Ca^2+^ event Δ*F*/*F* traces. *Left panels*: Plots of the Δ*F*/*F* traces of localized (blue) and propagative (orange) Ca^2+^ events. Traces have been aligned to their onset times and overlaid, with the mean profiles (bold line) and standard deviations (transparent shading) indicated. *Right panels*: Truncated violin plots quantifying the calculated area under the curve of the plotted Ca^2+^ event Δ*F*/*F* traces. ****p < 0.0001; Mann–Whitney test, two‐tailed. Cohorts (localized [Local], propagative [Prop]), ROUT 1% outlier exclusion used. (G) Soma‐situated Ca^2+^ events, *n* = [73, 72]. (H) Process‐situated Ca^2+^ events, *n* = [2005, 347]. (I–N) Truncated violin plots quantifying the temporal and spatial characteristics of microglial Ca^2+^ events. ****p < 0.0001, *p < 0.05; Mann–Whitney test, two‐tailed. Cohorts (localized [Local], propagative [Prop]), ROUT 1% outlier exclusion used. (I) Peak amplitude of soma‐situated Ca^2+^ events, *n* = [79, 82]. (J) Duration of soma‐situated Ca^2+^ events, *n* = [79, 80]. (K) Area of soma‐situated Ca^2+^ events, *n* = [67, 73]. (L) Peak amplitude of process‐situated Ca^2+^ events, *n* = [2124, 365]. (M) Duration of process‐situated Ca^2+^ events, *n* = [2184, 386]. (N) Area of process‐situated Ca^2+^ events, *n* = [1946, 335].

To capture all Ca^2+^ events occurring in a single microglia, we performed four‐dimensional in vivo two‐photon imaging for 10 min over five *Z*‐axis image planes with an acquisition rate of 2.5 frames/s (Figure S2, Video S1). Thus, the effective acquisition rate was 0.5 frames/s which affords a temporal resolution of 2 s and a non‐aliasing frequency maximum of 0.25 Hz as dictated by the Nyquist‐Shannon sampling theorem. The event‐based analysis of this microglial Ca^2+^ imaging data extracted numerous distinct Ca^2+^ events with highly diverse spatial (Figure 1A,B, Video S2), amplitude, and duration (Figure 1C) characteristics.

To establish an analysis framework for extracted Ca^2+^ events, we first examined Ca^2+^ event frequency to identify parameters suitable for categorization. Through this, we found the parameters of Ca^2+^ event location (soma vs. process) and propagation/spreading propensity (localized vs. propagative) presented as meaningful Ca^2+^ event categories. Initially, we observed Ca^2+^ events were significantly more frequent in the processes as compared to the soma (Figure 1D). Subsequently, we observed soma‐situated Ca^2+^ events had no significant differences in their likelihood to be localized or propagative (Figure 1E), whereas process‐situated Ca^2+^ events were significantly more likely to be localized (Figure 1F).

Having established these categories of Ca^2+^ events, we proceeded to more closely characterize their temporal dynamics. As a first step, we aligned the ΔF/F traces of all Ca^2+^ events to their onset times to generate average Ca^2+^ event plot profiles for each category (Figure 1G,H). Area under the curve (AUC) analysis of these plot profiles revealed significantly higher AUC values for propagative Ca^2+^ events compared to localized Ca^2+^ events, in both the soma (Figure 1G) and processes (Figure 1H). Since this AUC data is the product of Ca^2+^ event peak amplitude and duration, we next examined these parameters separately. Peak amplitudes were significantly higher for propagative Ca^2+^ events compared to localized Ca^2+^ events in the soma (Figure 1I), but were not significantly different in the processes (Figure 1L). Durations were significantly longer for propagative Ca^2+^ events compared to localized Ca^2+^ events in both the soma and processes (Figure 1J,M).

Finally, we characterized the spatial dynamics of these categories of Ca^2+^ events. To do this, we cumulatively summed the participating subcellular areas for each event. According to this criterion, subcellular areas were geographically contiguous with each other and exhibited Ca^2+^ activity at some point during the extracted Ca^2+^ event but not necessarily throughout the entirety of the event duration. Unsurprisingly, overall areas were significantly larger for propagative Ca^2+^ events compared to localized Ca^2+^ events in both the soma and processes (Figure 1K,N).

### 2.2 Directionality Based Characterization of Propagative Microglial Ca^2+^ Events

**FIGURE 2:**
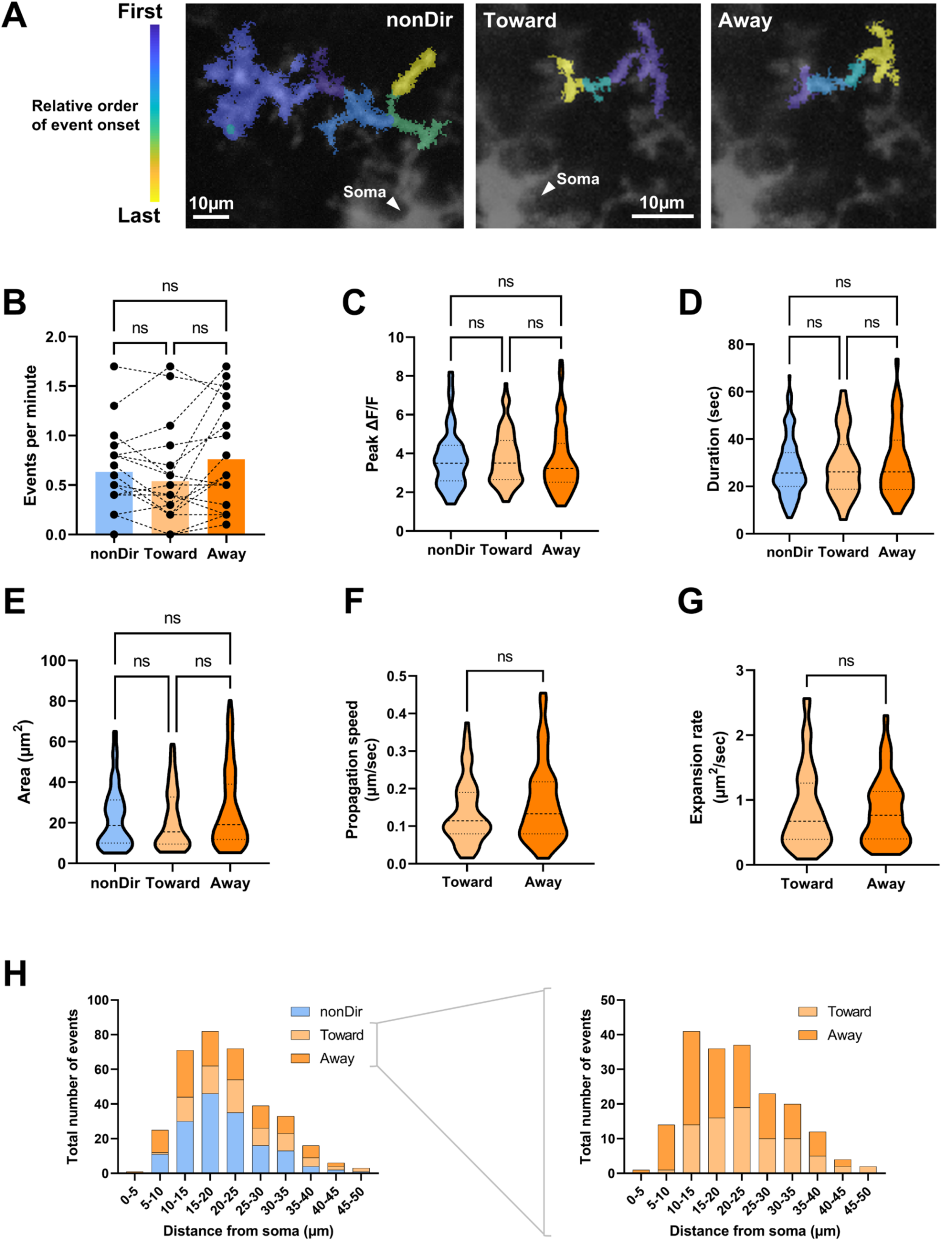
Directionality‐related characterization of process‐situated propagative microglial Ca^2+^ events. (A) Representative propagative Ca^2+^ events originating in the same microglial process but exhibiting different tendencies in their propagative direction—no overall directional tendency (left panel, “nonDir”), propagate toward the soma (middle panel, “Toward”), or propagate away from the soma (right panel, “Away”). Scale bar = 10 μm, arrowhead indicates microglial soma. Note that the “Toward” and “Away” examples come from the same microglia, whereas the “nonDir” example comes from a different microglia. (B) Dot plots quantifying the frequency of process‐situated microglial Ca^2+^ events with a propagative direction that is “nonDir,” “Toward,” or “Away” relative to the microglial soma. Each triplet of data points represents one microglial cell, *n* = 18. Columns indicate cohort means. ns, not statistically significant; Friedman's test with post hoc Dunn's multiple comparisons test, two‐tailed. (C–E) Truncated violin plots quantifying the temporal and spatial characteristics of process‐situated microglial Ca^2+^ events. ns, not statistically significant; –Wallis test with post hoc Dunn's multiple comparisons test, two‐tailed. Cohorts (nonDir, Toward, Away), ROUT 1% outlier exclusion used. (C) Peak amplitude of Ca^2+^ events, *n* = [147, 75, 101]. (D) Duration of Ca^2+^ events, *n* = [157, 79, 110]. (E) Area of soma‐situated Ca^2+^ events, *n* = [151, 70, 101]. (F, G) Truncated violin plots quantifying the spatio‐temporal characteristics of process‐situated propagative microglial Ca^2+^ events. ns, not statistically significant; Mann–Whitney test, two‐tailed. Cohorts (Toward, Away), ROUT 1% outlier exclusion used. (F) Propagation speed of Ca^2+^ events, *n* = [71, 106]. (G) Area expansion rate of Ca^2+^ events, *n* = [72, 97]. (H) Histograms quantifying the total number of process‐situated microglial Ca^2+^ events originating at various distances away from the microglial soma. Ca^2+^ events have been categorized based on whether the directional tendency of their propagation shows no overall directional tendency “nonDir” (blue) or is “Toward” (light orange) or “Away” (dark orange) from the microglial soma. Ca^2+^ events are pooled from 18 microglia (left histogram). “Toward” and “Away” Ca^2+^ events have also been re‐plotted alone in the right histogram for clearer visualization.

In neurons, the flow of electrical activity conveys information from one subcellular region to another. Analogously, propagative Ca^2+^ events may serve a similar function in microglia. In this case, the directionality of Ca^2+^ event propagation may be informative in decoding how microglia integrate and compute information about the surrounding environment. Thus, we characterized propagative process‐situated Ca^2+^ events in more detail, categorizing them as having no overall directional tendency “nonDir,” propagating toward the soma “Toward,” or propagating away from the soma “Away” (Figure 2A). We observed no significant differences in the temporal and spatial dynamics of these categories in terms of their frequency (Figure 2B), peak amplitude (Figure 2C), duration (Figure 2D), and overall area (Figure 2E). Note that we excluded any Ca^2+^ events that intruded into the soma from analysis to ensure unambiguous categorization (Figure S4).

Subsequently, we compared the spatio‐temporal dynamics of “Toward” and “Away” propagative events by examining the parameters of average propagation speed and average rate of area increase. When calculating the average propagation speed, we used the continuous distance along the microglial process over which a Ca^2+^ event progressed. The start and end points of this distance corresponded to the centroid of fluorescence distribution of an event's terminal locations (onset/starting and offset/ending sites). Defined this way, average propagation speed was not significantly different between “Toward” and “Away” Ca^2+^ events (Figure 2F). Subsequently, we examined whether Ca^2+^ event propagation tended to proceed by the Ca^2+^ event area expanding into new territory or by the Ca^2+^ event area remaining relatively constant in size whilst progressively traveling into new territory. Thus, we calculated the average rate of Ca^2+^ event area increase (i.e., expansion rate), using the Ca^2+^‐active areas observed at an event's onset/starting and offset/ending. Defined this way, expansion rate was not significantly different between “Toward” and “Away” Ca^2+^ events (Figure 2G). Finally, we examined whether there was any relationship between the frequencies of process‐situated “Local,” “Toward,” and “Away” Ca^2+^ events and the relative proximity of their onset location to the microglial soma (Figure 2H). The frequencies for all Ca^2+^ event categories appeared to follow a similar trend, peaking at a distance of 10–15 μM away from the microglial soma.

### 2.3 Compartmentalization of Microglial Ca^2+^ Events Corresponds to Microglial Branch Points

**FIGURE 3:**
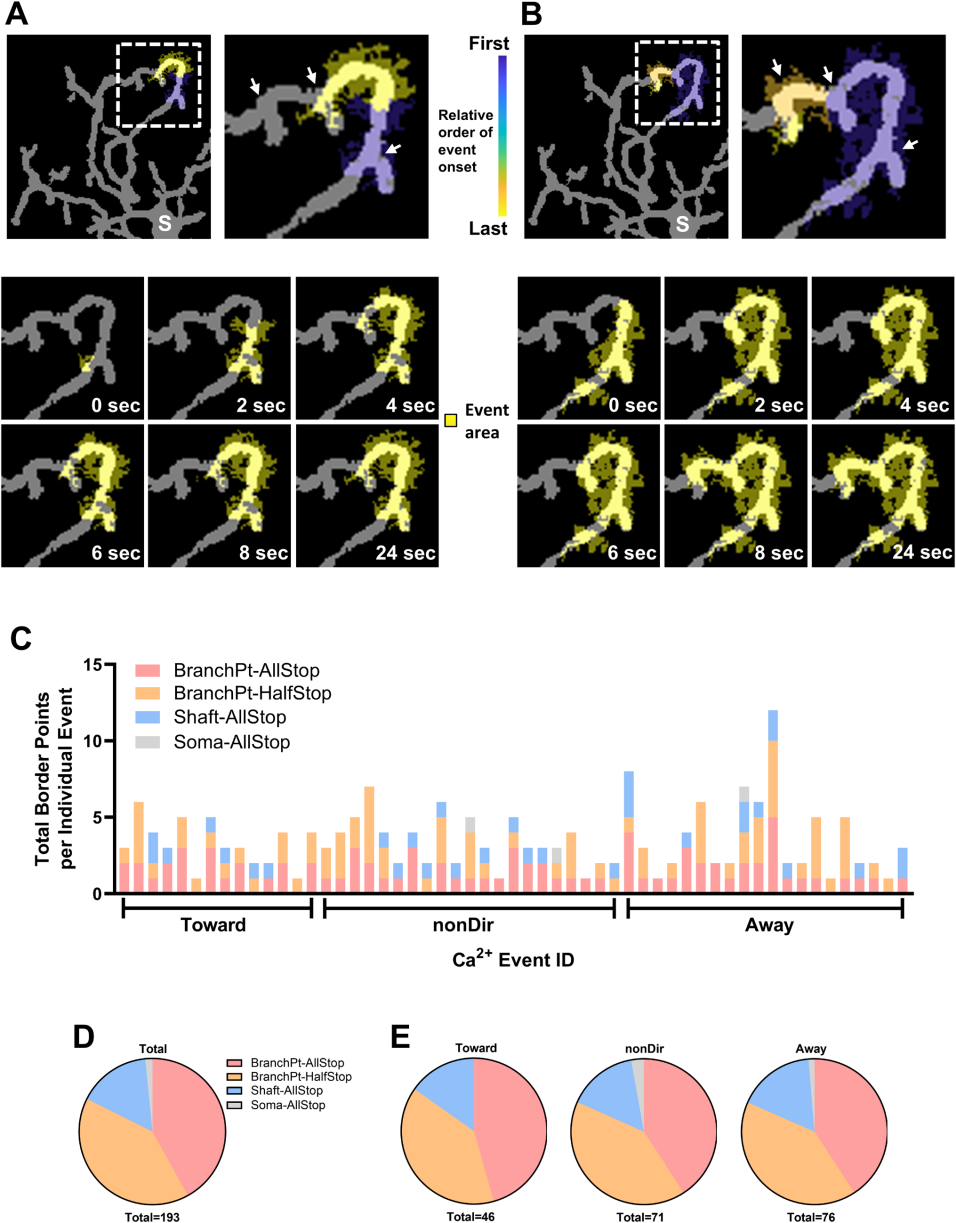
Process‐situated microglial Ca^2+^ events may exhibit a “stop and go” pattern of propagation. (A, B) Two separate examples of a process‐situated microglial Ca^2+^ event exhibiting a “stop and go” pattern of propagation. Note that both examples take place in the same microglial process segment but at different times. *Top panels*: The Ca^2+^ event visualized as its AQuA‐derived spatio‐temporal footprint (color scale) over its entire lifetime. The top‐right panel is a magnified view of the area demarcated by the dashed square in the top‐left panel. The soma (S) and microglial process branch points (arrows) are indicated. *Bottom panels*: The same Ca^2+^ event visualized as its AQuA‐derived spatial footprint (yellow shading) at 0, 2, 4, 6, 8, and 24 s after its onset. All panels are a magnified view of the area demarcated by the dashed square in the top‐left panel. Note that the Ca^2+^ event's spatial footprint does not change across multiple panels, indicative of a “stop and go” propagation pattern and tends to be bordered by microglial process branch points. (C) Bar graph quantifying the number and types of border points for individual process‐situated propagative Ca^2+^ events during the first encounter of their wavefront with a microglial branch point. Each bar represents an individual Ca^2+^ event. Events are grouped according to the directional tendency of their propagation: “Toward” or “Away” from the microglial soma, or no overall direction “nonDir.” Border points are defined as locations where the Ca^2+^ event's wavefront paused for ≥ 2 s, with four types observed: (1) BranchPt‐AllStop (Pink), complete pause at the microglial branch point with no entry into either daughter branch; (2) BranchPt‐HalfStop (Orange), partial pause at the microglial branch point with entry into one daughter branch but exclusion from the other; (3) Shaft‐AllStop (Blue), pause in the middle of a microglial process; (4) Soma‐AllStop (Gray), pause at the soma boundary. A total of 55 Ca^2+^ events (top ~20% by Δ pixel area) were analyzed, with all events (*n* = 55/55) exhibiting STOP behavior at a microglial branch point (BranchPt‐AllStop or BranchPt‐HalfStop), indicating a significant constraint on Ca^2+^ propagation. Binomial exact test (null hypothesis = 50% chance), two‐tailed. (D, E) Pie charts showing the proportional breakdown of border point categories pooled from the Ca^2+^ events analyzed in (C). Color key: BranchPt‐AllStop (pink), BranchPt‐HalfStop (orange), Shaft‐AllStop (blue), and Soma‐AllStop (gray). (D) A total of 193 border points pooled from all *n* = 55 Ca^2+^ events. (E) Border points pooled from Ca^2+^ events as sub‐categorized by the directional tendency of their propagation, Direction (total border points, Ca^2+^ events). Toward [46, 14] the soma; Away [76, 20] from the soma; nonDir [71, 21] no overall directional bias.

In neurons, spine and dendrite morphology have significant influence over the filtering and thresholding of electrical activity. Analogously, microglial Ca^2+^ activity is likely subject to various filtering and thresholding mechanisms given that its behavior can be neatly categorized into localized and propagative Ca^2+^ events. Intuitively, higher peak amplitude should correlate with increased likelihood for propagation. Whilst such reasoning appears to hold true for soma‐situated Ca^2+^ activity (Figure 1I), it is contradicted by process‐situated Ca^2+^ activity (Figure 1L). Considering the complex yet non‐random morphology of ramified microglial processes, we examined whether their branch points could be correlated with the compartmentalization of process‐situated Ca^2+^ events. Qualitatively, we observed several examples where a propagative Ca^2+^ event would momentarily pause (STOP) at a microglial process branch point before resuming (GO) its propagation (Figure 3A,B).

To more quantitatively assess this STOP‐GO behavior, we ranked propagative process‐situated Ca^2+^ events by their change in pixel area (Δ area = area at maximal spatial extent—area at onset) and selected the top ~20% (Δ area > 300 pixels) for further analysis. We presumed Ca^2+^ events that spread over a larger area were more likely to travel through at least one microglial branch point. From the initial 66 identified Ca^2+^ events, we excluded 11 from further analysis because they entered highly motile regions of the microglia, specifically the process tips, for which it was not possible to reconstruct a morphological map that remained accurate for the entire observation period (Figure S5).

For each of the remaining 55 Ca^2+^ events, we identified the time‐point at which the Ca^2+^ wavefront intersected with a microglial branch point. STOP behavior was defined if the wavefront remained fully bounded (BranchPt‐AllStop—no propagation into either daughter branch) or partially bounded (BranchPt‐HalfStop—propagation into one daughter branch but exclusion from the other) at the microglial branch point for at least 2 s (two consecutive frames, 0.5 Hz effective sampling rate). Throughout the period in which the Ca^2+^ wavefront maintained this same STOP state at the microglial branch point, we also documented all other boundary points where the wavefront's remaining perimeter exhibited STOP behavior. We considered four boundary point categories: BranchPt‐AllStop, BranchPt‐HalfStop, Shaft‐AllStop (boundary in the middle of a microglial process), Soma‐AllStop (boundary at the soma border).

Strikingly, all 55 Ca^2+^ events exhibited STOP behavior at a microglial branch point either as BranchPt‐AllStop or BranchPt‐HalfStop (Figure 3C). Accordingly, microglial branch points were strongly associated with the compartmentalization of process‐situated Ca^2+^ events, with a statistically significant higher probability of compartmentalization at these sites above chance expectation. Notably, of these 55 Ca^2+^ events, 24 exhibited STOP‐GO behavior, eventually resuming their propagation past the original microglial branch point (Figure S6). Separately, Ca^2+^ compartmentalization behavior did not seem to change depending on propagation direction (Figure 3D,E).

### 2.4 Physiological Microglial Ca^2+^ Activity Is Significantly Underpinned by Purinergic Signaling

**FIGURE 4:**
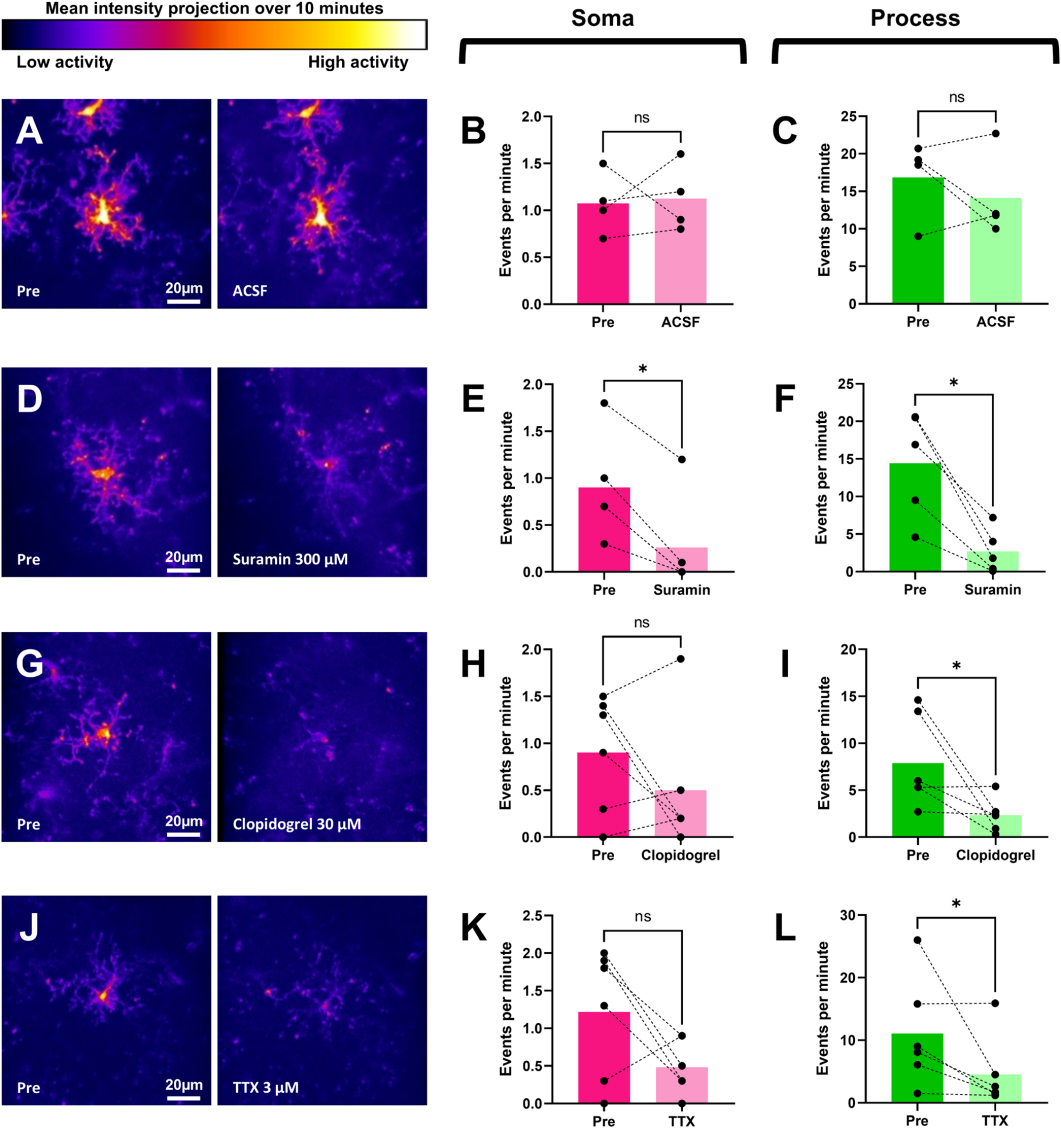
Ca^2+^ activity in physiological microglia is significantly driven by P2Y12 signaling and putative neuronal activity. (A–L) Microglial Ca^2+^ activity is (A–C) unaffected by ACSF exchange but reduced by application of (D–F) 300 μM suramin, a broad spectrum P2 antagonist; (G–I) 30 μM clopidogrel, a P2Y12‐specific antagonist; and (J–L) 3 μM TTX. Given microglia do not express TTX‐sensitive Na^+^ channels, the effect of TTX is attributed to it mediating a reduction in neuronal activity. (A, D, G, J) Mean intensity projection of GCaMP6 fluorescence observed pre‐ (left panel) and 10‐min post‐ (right panel) drug application. The color scale is indicative of the average level of Ca^2+^ activity exhibited within the various subcellular regions of the microglia during 10‐min duration of both observation windows. Scale bar = 20 μm. (B, C, E, F, H, I, K, L) Dot plots quantifying the frequency of microglial Ca^2+^ events as classified by whether they are soma‐situated (pink palette) or process situated (green palette). Each pair of data points represents one microglia cell. Columns indicate cohort means. *p < 0.05; Wilcoxon's matched‐pairs sign ranked test, one‐tailed. (B, C) Pre‐ versus post‐ACSF exchange, *n* = 4; (E, F) pre‐ versus post‐suramin, *n* = 5; (H, I) Pre‐ versus post‐clopidogrel, *n* = 6; (K, L) pre‐ versus post‐TTX, *n* = 6.

The distinct dynamics between soma‐situated and process‐situated Ca^2+^ events suggest that different regulatory signaling pathways may govern activity at these sites. Thus, Ca^2+^ activity at these sites may respond differently to changes in the surrounding environment. Given the sensitivity of microglia to extracellular purinergic ligands, we compared Ca^2+^ dynamics before and after direct application of suramin to the exposed but intact brain surface of awake mice (Figure S7). Note that suramin is a broad spectrum purinergic P2 receptor antagonist that is not typically able to cross the cell membrane. In separate experiments, we checked whether the drug application procedure itself affected microglial Ca^2+^ activity dynamics by exchanging ACSF over the exposed brain surface. As expected, ACSF exchange had no significant effect on the frequencies of both soma‐situated and process‐situated Ca^2+^ events (Figure 4A–C). In contrast, suramin application significantly reduced the frequencies of both soma‐situated and process‐situated Ca^2+^ events (Figure 4D–F).

Whilst microglia express a diverse array of purinergic P2 receptors, the P2Y12 subtype may be especially functionally significant. It is only expressed by ramified microglia under strictly physiological conditions, has enriched expression in microglial processes, and is functionally linked to microglial process motility (Haynes et al. 2006; Sipe et al. 2016). Thus, we compared microglial Ca^2+^ activity before and after the application of the P2Y12‐specific antagonist clopidogrel to the exposed but intact brain surface of awake mice. Intriguingly, this had no significant effect on the frequency of soma‐situated Ca^2+^ events but significantly reduced the frequency of process‐situated Ca^2+^ events (Figure 4G–I).

### 2.5 Putative Neuronal Activity Influences the Basal Level of Physiological Microglial Ca^2+^ Activity

Previous reports and our data indicate that microglial Ca^2+^ activity is significantly driven by extracellular purinergic input (Eichhoff et al. 2011; Moller et al. 2000; Pozner et al. 2015). Given the close interactions between microglia and neurons, it is possible neuronal activity is a major source of this. Indeed, the dynamics of repeated transient contact between microglial processes and neuronal synapses and somas, as well as microglial process extension‐retraction and Ca^2+^ activity during environmental surveillance, are influenced by the ambient level of neuronal activity (Eyo et al. 2014; Li et al. 2012; Umpierre et al. 2020; Wake et al. 2009). To examine how microglial Ca^2+^ activity may be influenced by neuronal activity, we compared its dynamics before and after direct application of TTX to the exposed but intact brain surface of awake mice. Interestingly, this significantly reduced the frequency of process‐situated, but not soma‐situated, Ca^2+^ events (Figure 4J–L).

## 3 Discussion

Using an unbiased and quantitative approach, this study revealed that microglial Ca^2+^ activity exhibits spatio‐temporally diverse dynamics. Whilst most Ca^2+^ events remain localized, a substantial number propagate between subcellular regions. Notably, this Ca^2+^ propagation appears neither passive nor random. It exhibits strong directional bias and, at process branch points, often only enters select processes whilst bypassing others. Such behavior implies active regulation by extracellular and intracellular cues, which have yet to be fully characterized.

### 3.1 Mechanisms Regulating Microglial Ca^2+^ Activity

Given their well‐known association with microglial Ca^2+^ activity, extracellular ATP/UDP are likely among its key regulators (Ferrari et al. 1996; Moller et al. 2000; Walz et al. 1993). Indeed, microglia express both P2X (ionotropic) and P2Y (metabotropic) purinergic receptors at high levels (Calovi et al. 2019). Early studies in cultured microglia demonstrated that extracellular ATP elicits a large Ca^2+^ response, driven by distinct ionotropic and metabotropic signaling components (Ferrari et al. 1996; Moller et al. 2000; Walz et al. 1993). Later in vivo studies confirmed similar robust Ca^2+^ responses to ATP and UDP, as well as selective P2X and P2Y receptor agonists (Eichhoff et al. 2011; Pozner et al. 2015). Importantly, these signaling pathways are implicated in modulating microglial Ca^2+^ activity in response to changes in the surrounding environment (e.g., LPS activation and laser burns), aging and disease (e.g., stroke and epilepsy) (Liu et al. 2021; Olmedillas Del Moral et al. 2020; Pozner et al. 2015; Umpierre et al. 2020). In particular, P2Y12 and P2Y6 receptor signaling have been specifically identified as primary drivers of altered microglial Ca^2+^ activity in mouse models of Alexander's disease and epileptogenesis (Saito et al. 2024; Umpierre et al. 2024).

The role of P2Y12 signaling may be especially important given expression of this receptor subtype is almost completely absent from all CNS cell types except for physiological microglia. P2Y12 receptors are G‐inhibitory protein‐coupled receptors and their activation markedly increases Ca^2+^ activity in physiological microglia (Ma et al. 2024), largely through downstream PLC‐IP_3_ signaling (De Simone et al. 2010; Irino et al. 2008; Obal and Krueger 2001). Although P2Y12 receptors are widely expressed at both the soma and processes of microglia (Haynes et al. 2006), pharmacological experiments in this study suggest their contribution to microglial Ca^2+^ activity may differ between these two sub‐regions. The event‐based analysis enabled unbiased deconvolution of microglial Ca^2+^ activity into discrete soma‐situated or process‐situated Ca^2+^ events. Broad purinergic antagonism with suramin significantly reduced Ca^2+^ activity in both sub‐regions (Figure 4E,F), whereas selective P2Y12 antagonism with clopidogrel only suppressed process‐situated Ca^2+^ activity (Figure 4H,I). Taken together, these results suggest that P2Y12 signaling is a primary regulator of process‐situated microglial Ca^2+^ activity, whilst other purinergic receptors play a more significant role in the soma.

The constrained spatial dynamics of process‐situated Ca^2+^ activity may also be related to P2Y12 signaling. Since P2Y12/PLC‐IP_3_ signaling mediates Ca^2+^ release from the endoplasmic reticulum (ER), the ER's structure itself may dictate its spatial dynamics. In neuronal dendrites, the ER can support long‐distance Ca^2+^ propagation via Ca^2+^‐induced Ca^2+^ release, owing to its continuous distribution and highly reticulated structure (Berridge 1998, 2002). Microglial processes also contain ER (El Hajj et al. 2019), however, quantification of its continuity/distribution is far less comprehensive than for neurons. Nevertheless, electron microscopy data from a large scale study of human microglia‐like cells is consistent with a non‐uniform ER distribution (Shapson‐Coe et al. 2024). Moreover, transmission electron microscopy from ultrathin mouse brain sections has quantified process‐like structures at the microglial soma that are less than 300 nm thick and unambiguously devoid of ER and Golgi bodies (Savage et al. 2019). Taken together, these prior studies suggest that the ER might adopt a fragmented and discontinuous distribution in some microglial processes. This structural characteristic could underlie the highly localized nature of process‐situated Ca^2+^ events and their restricted propagation to specific processes, as observed in this study (Figures 2 and 3).

### 3.2 Functional Significance of Microglial Ca^2+^ Activity Dynamics

A characteristic feature of ramified physiological microglia is that their processes are continuously motile, dynamically responding to changes in the surrounding environment (Nimmerjahn et al. 2005). Furthermore, the actions of each process are highly independent from each other and the soma. For example, in a single microglia, the tips of one process may engage in phagocytosis of dead cells (Sierra et al. 2010) whilst other processes continue to surveil the environment and/or modulate neuronal activity (Kamei and Okabe 2023). Thus, the highly localized nature of process‐situated Ca^2+^ events and their restricted propagation to specific processes, as observed in this study (Figures 2 and 3), are consistent with this independent process behavior. This observation refines previous reports correlating microglial process extension/retraction with changes in Ca^2+^ activity (Pozner et al. 2015; Umpierre et al. 2020).

It is also noteworthy that microglial Ca^2+^ activity was similarly affected by putative neuronal inhibition with TTX and selective P2Y12 receptor antagonism with clopidogrel. Process‐situated Ca^2+^ events were significantly reduced, whereas soma‐situated Ca^2+^ events were less affected (Figure 4G–O). This suggests that microglial responsiveness to changes in neuronal activity is greatly influenced by P2Y12 signaling, at least in some contexts. Intriguingly, Cserep et al. (2020) have identified specialized junctions between microglial processes and the neuronal soma that contain nanostructures optimized for purinergic signaling. The formation of these junctions requires both neuronal activity and P2Y12 receptor function and appears to regulate neuronal Ca^2+^ loading, highlighting a potential mechanism by which individual microglia can provide targeted neuroprotection to multiple neighboring neurons.

On the other hand, the results of these pharmacology experiments should be interpreted with caution. Umpierre et al. (2020) previously reported increased microglial Ca^2+^ activity following selective inhibition of excitatory CaMKII‐expressing neurons using Gi‐DREADDs. This discrepancy may reflect differences in experimental conditions. Umpierre and colleagues performed their experiments in chronic preparations, whereas our measurements were taken acutely after cranial window surgery, a period during which both neuronal activity and microglial Ca^2+^ signals are elevated (see also Figure S3). This is thought to reflect a more inflammatory environment which would influence microglial behavior. Indeed, microglia perform seemingly paradoxical roles—both pruning synapses and promoting their formation—depending on local environmental cues (Miyamoto et al. 2016; Schafer et al. 2012).

Future studies are needed to more precisely dissect the relationship between neuronal activity and microglial Ca^2+^ dynamics. Ideally, neuronal and microglial Ca^2+^ activity should be imaged simultaneously, for example using GCaMP8f in neurons and jRGECO in microglia, as the fast kinetics of GCaMP8f are required to accurately capture neuronal events. However, there are significant technical challenges to expressing red‐shifted GECIs in glial cells, where protein aggregation remains a known issue (Dana et al. 2016; Shen et al. 2018). We also note ongoing debate about whether microglia express TTX‐sensitive voltage gated Na^+^ channels. Whilst physiological in vivo microglia (and astrocytes) are generally thought not to express these channels (Zhang et al. 2014), activated or in vitro cultured microglia can (Hossain et al. 2017), complicating mechanistic interpretation. Overall, this study is not intended as a comprehensive dissection of microglial signaling pathways but rather highlights a reliable, sensitive, and time‐efficient approach for quantifying subcellular changes in microglial Ca^2+^ activity, which is particularly relevant given their complex and ramified morphology.

## 4 Materials and Methods

### 4.1 Animals

All procedures were performed at the National Institute for Physiological Sciences, which is a constituent institute of the National Institutes for Natural Sciences. All procedures were approved by the Animal Care and Use Committee of the National Institutes of Natural Sciences. All procedures were performed in accordance with all relevant guidelines and regulations, including National Institutes of Health guidelines.

The Iba1‐GCaMP6 mouse line was established by crossing Iba1‐tTA mice with tetO‐GCaMP6 mice. The Iba1‐tTA mice were a kind gift from Prof. Kenji Tanaka (Keio University, Tokyo, Japan). In these mice, the ionized Ca^2+^ binding adapter molecule 1 (Iba1) promoter, which is only active in microglia, drives the expression of the tetracycline trans‐activator (tTA) (Tanaka et al. 2012). The tetO‐GCaMP6 mice were provided by RIKEN BRC through the National BioResource Project of the MEXT/AMED. In these mice, GCaMP6 expression is driven by the tetracycline response element (tetO) promoter (Ohkura et al. 2012). Thus, in Iba1‐tTA mice, the Iba1 promoter, through coupling mediated by the tetracycline conditional (tetO‐tTA) expression system, drives expression of GCaMP6 exclusively in microglia. Note that all mice were backgrounded on the C57Bl/6J strain.

Male mice were used for all experiments and were 8–10 weeks old at the time of surgery. After weaning and sex separation, mice were housed in groups of four–six animals per cage under a 12‐h light–dark cycle with ad libitum access to food and water. Prior to weaning, mice were raised on standard rodent chow supplemented with 100 mg/kg doxycycline (Oriental Yeast Co. Ltd., Tokyo, Japan) to avoid the possibility of GCaMP6 expression causing off‐target developmental defects. Post‐weaning, mice were then switched to doxycycline‐free standard rodent chow.

### 4.2 Surgery

The surgical procedure to prepare mice for in vivo brain imaging was performed over 2 days. On the first day, a custom‐made metal head‐plate was attached to the skull. On the second day, a cranial window was made over the left primary motor cortex (M1), centered 0.2 mm anterior and 1.0 mm lateral to Bregma.

For the head‐plate attachment surgery, mice were anesthetized using ketamine (7.4 mg per kg bodyweight, i.p.) and xylazine (10 mg per kg bodyweight, i.p.). Topical application of 2% xylocaine jelly (AstraZeneca plc, Cambridge, UK) to the scalp was used for further pain management. Following this, the scalp was shaved and sterilized with 70% (w/vol) ethanol. The skull was then exposed and cleaned before attaching a custom‐made metal head‐plate with dental cement (G‐Cem One, GC Corporation, Tokyo, Japan). After curing, the dental cement and any exposed surfaces of the skull were coated with an acrylic‐based dental adhesive resin (Super bond; Sun Medical, Shiga, Japan).

For the cranial window surgery, mice were anesthetized using isoflurane (4% for induction, 1.0%–1.5% for maintenance). A craniotomy was then performed by demarcating and thinning its borders using a dental drill before removing the marked skull piece using a fine surgical hook (Narishige, Tokyo, Japan). Following this, a double‐layered glass window was secured into the craniotomy with UV craft resin (UVR, Kiyohara, Japan). Note that the two layers of the glass window were bonded together prior use with UV cured optic resin (Norland Optical Adhesive 61; Norland Products Inc., New Jersey, USA). For imaging only experiments, craniotomies were circular with a diameter of 2 mm. Dimensions of the corresponding glass window were 2 mm diameter (bottom layer) and 4 mm diameter (top layer), thus enabling a waterproof seal with the bordering skull. For pharmacology experiments, craniotomies were rectangular with an anteroposterior length of 3 mm and a lateral length of 2 mm. Dimensions of the corresponding glass window were 2 mm × 2 mm (bottom layer) and 3 mm anteroposterior × 4 mm (top layer), thus part of the brain surface remained exposed once the window was secured in place.

### 4.3 Pharmacological Agents

For the pharmacology experiments, all drugs were administered by direct application to the exposed brain surface. All drugs were diluted in artificial cerebrospinal fluid (ACSF: 126 mM NaCl, 2 mM KCl, 2 mM CaCl_2_, 24 mM NaHCO_3_, 1.2 mM NaH_2_PO_4_, 1.3 mM MgSO_4_, and 10 mM glucose). Care was taken to ensure that the exposed brain surface was always covered with ACSF. The drugs used were as follows: Tetrodotoxin (3 μM; Wako, Japan), Suramin (300 μM; Sigma, USA), and Clopidogrel (30 μM; Tokyo Chemical Industry, Japan).

### 4.4 Four‐Dimensional Two‐Photon Ca^2+^ Imaging in Awake Brain

In vivo two‐photon imaging was performed using a Nikon A1 microscope (Nikon, Tokyo, Japan), a water‐immersion objective lens (×25, numerical aperture 1.10; Nikon, Tokyo, Japan), and a Ti‐sapphire laser (Mai Tai HP, Spectra‐Physics, California, USA) operating at 920 nm wavelength. Four‐dimensional images (512 × 512 pixel^2^, 0.25 μm/pixel, five *Z*‐planes separated by 3 μm) were acquired over 10 min for acute‐state imaging or 30 min for chronic‐state imaging at 2.5 frame/s using resonant scanning (*XY*‐axes) and a piezo nanopositioning system (*Z*‐axis; Nano‐Drive, Mao City Labs, Wisconsin, USA). A 560 nm dichroic mirror and a 500–550 nm band‐pass emission filter were used to ensure selective detection of GCaMP6 fluorescence.

All imaging was performed in awake mice. For acute‐state imaging‐only and pharmacology experiments, imaging was performed 30–60 min after surgery to allow mice to recover from any residual effects of isoflurane anesthesia. For chronic‐state imaging‐only experiments, imaging was performed several weeks after surgery.

### 4.5 Image Analysis of Microglial Ca^2+^ Activity

Acquired four‐dimensional images were registered in MATLAB (R2017b; MathWorks Inc., Massachusetts, USA) using the ECC image alignment algorithm (https://www.mathworks.com/matlabcentral/fileexchange/27253‐ecc‐image‐alignment‐algorithm‐image‐registration) (Evangelidis and Psarakis 2008). Mean intensity projections were used to reconstruct microglial morphology. Alternatively, max intensity projections were used when the level of fluorescence activity was very low. Regions of the microglia which remained stationary throughout the 10‐min acquisition period (stable area) were identified by comparing intensity projections calculated from the first and last 2 min of the acquisition period.

Discrete Ca^2+^ events were extracted from GCaMP6 fluorescence using the AQuA analysis pipeline in MATLAB as previously described (Wang et al. 2019). The use of AQuA for analyzing microglial Ca^2+^ activity was validated against established ROI‐based analysis methods (Figure S9). Each event was categorized as “soma‐situated” or “process‐situated” based on its point of origin. Subsequently, events were classified as “localized” or “propagative” based on the change in pixel area (0.25 μm/pixel) between the event's onset and the time‐point at which the event reached its maximal spatial extent. “Soma‐situated” events were classified as “propagative” if the increase in pixel area exceeded 167 pixels. “Process‐situated” events were classified as “propagative” if the increase in pixel area exceeded 79 pixels. The directionality of “propagative” events was determined using AQuA‐derived “chgToward” and “chgAway” scores, which track the frame‐by‐frame movement of event‐related pixels relative to a specified landmark (the soma). A directional index was calculated as the “chgToward/chgAway” ratio, with events classified as “toward” (ratio > 3), “away” (ratio < 1/3), or “nonDir” (no overall directional tendency, 1/3 < ratio < 3). The threshold values for “localized” versus “propagative” and “toward” versus “away” versus “nonDir” were all determined using receiver operating characteristic (ROC) analysis based on manual expert labeling of a random subset of events.

### 4.6 Immunohistochemistry

Mice were deeply anesthetized with ketamine (0.13 mg/g, i.p.) and xylazine (0.01 mg/g, i.p.) and perfused with 4% paraformaldehyde (PFA). Whole brains were harvested, post‐fixed (4% PFA, 1 day, 4°C), and sectioned (coronal, 50 μm thick) using a vibratome (VT1000S; Leica Biosystems, Wetzlar, Germany). Sections were incubated in blocking solution (5% bovine serum albumin and 0.5% Triton X‐100 in 0.1 M phosphate buffer, 30 min, room temperature), washed with phosphate buffered saline (PBS), and incubated in primary antibodies (overnight, 4°C) for GFP (1:1000 dilution, mouse anti‐GFP MAB3580; Millipore, USA) and Iba1 (1:1000 dilution, rabbit anti‐Iba1 019‐19741; Wako, Osaka, Japan). The next day, sections were washed with PBS and incubated in secondary antibodies (2 h, room temperature) for mouse/anti‐GFP (1:1000 dilution, Alexa Fluor 488 donkey anti‐mouse; Life Technologies, Carlsbad, USA) and rabbit/anti‐Iba1 (1:1000 dilution; Alexa Fluor 594 donkey anti‐rabbit; Life Technologies, Carlsbad, USA). Following this, sections were washed with PBS and coverslipped with fluorescence protecting mounting medium (Vectashield with DAPI, H‐1200; Vector Laboratories, California, USA). Sections were imaged (1024 × 1024 pixel^2^, 0.31 μm/pixel, 1 μm *Z*‐axis resolution) using a Nikon A1R confocal microscope (Nikon, Tokyo, Japan) and a water‐immersion objective (×40, numerical aperture 1.15; Nikon, Tokyo, Japan).

### 4.7 Data and Statistics

All statistical tests were performed using GraphPad Prism 9. In all cases, non‐parametric statistical tests were used with the alpha significance level set to 0.05. Two‐tailed hypothesis testing was performed for Figures 1, 2, 3 and Figures S3 and S9. One‐tailed hypothesis testing was performed for Figure 4 and Figure S8.

## Author Contributions

H.H. and J.N. designed the study. H.H. and J.I. performed experiments. H.H. analyzed data. H.H., D.L.C., and J.N. wrote the paper.

## Funding

This work was supported by Japan Society for the Promotion of Science (16K19001, 18K14825, 21H05639, 21H03027, 24K02543, 24K02213, 17H01530, and 20H00500) and AMED under Grant Number 111049.

## Conflicts of Interest

The authors declare no conflicts of interest.

## Supporting information


**Figure S1:** GCaMP6 is exclusively expressed by microglia in transgenic Iba1‐GCaMP6 mice.

**Data S1:** Supporting information.

**Figure S2:** Schematics of the setup and procedure for in vivo two‐photon imaging of microglia.

**Figure S3:** Imaging and characterization of microglial Ca^2+^ in mice several weeks following cranial window surgery (chronic‐state).

**Figure S4:** Exclusion of process‐situated “Toward” category Ca^2+^ events from analysis.

**Figure S5:** Reconstruction process of the 2‐dimensional microglial morphology map used in Figure 3A,B.

**Figure S6:** Further characterization of STOP‐GO behavior by propagative process‐situated Ca^2+^ events.

**Figure S7:** Schematics of the setup and procedure for in vivo two‐photon imaging of microglia.

**Figure S8:** Sub‐categorization of process‐situated microglial Ca^2+^ events observed in Figure 4 pharmacology experiments as localized vs. propagative.

**Figure S9:** Validation of microglial Ca^2+^ event detection by the AQuA analysis pipeline.

**Video S1:** Ca^2+^ activity in individual microglial cells over 10 min visualized by changes in raw GCaMP6 fluorescence using in vivo two‐photon imaging in an awake mouse.

**Video S2:** Deconvolution of raw GCaMP6 fluorescence signals (from Video 1) into discrete Ca^2+^ events using the AQuA analysis pipeline.

## Data Availability

The data that support the findings of this study are available from the corresponding author upon reasonable request.
