# Supplementary figures and images for: Spatio‐Temporal Diversity of Calcium Activity in Microglia
Source: Glia. 2026 Jan 6;74(3):e70131. doi: 10.1002/glia.70131 (PMC12772526; doi:10.1002/glia.70131)

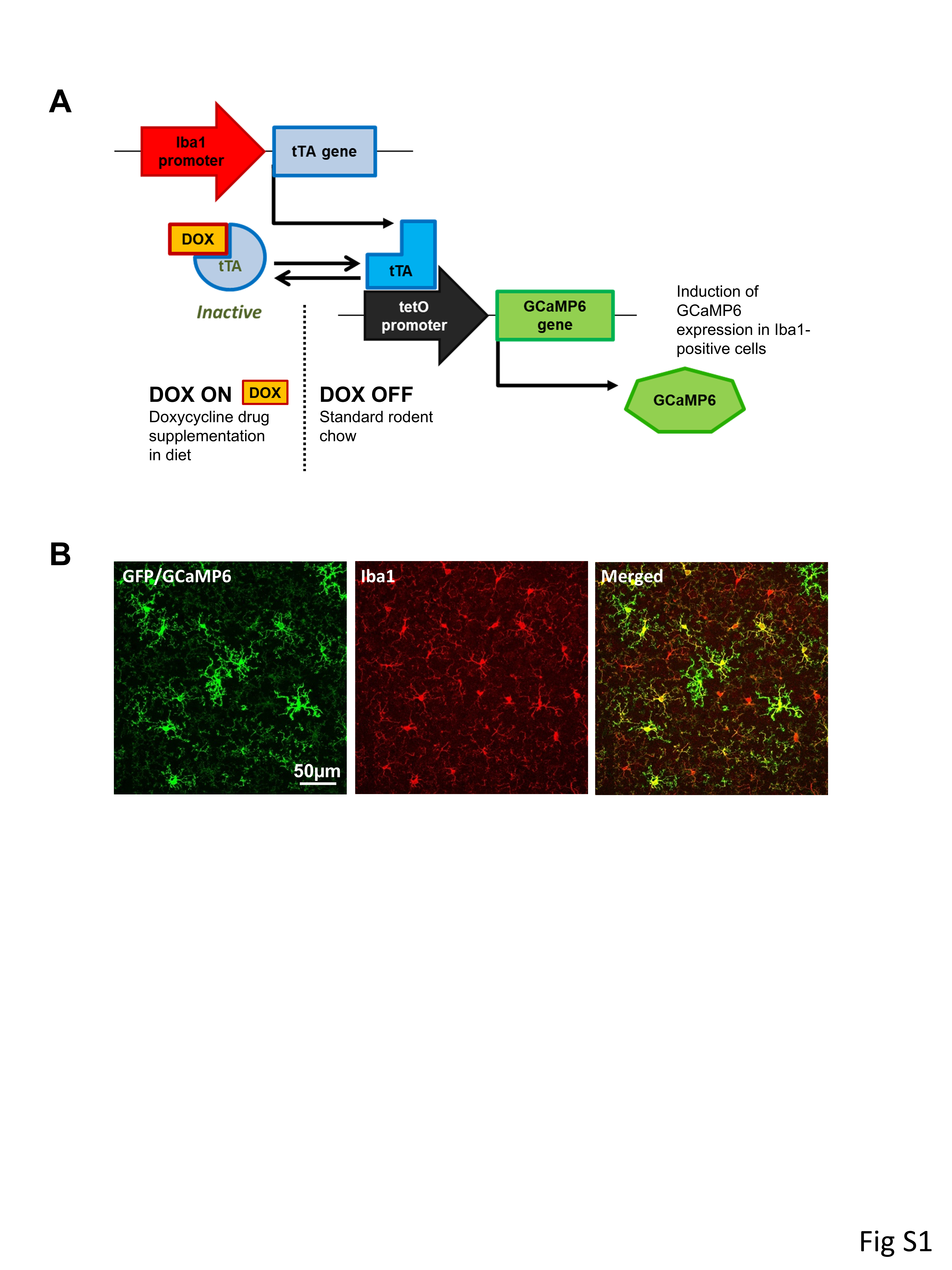

Supplement: Supplementary file 1 — Figure S1: GCaMP6 is exclusively expressed by microglia in transgenic Iba1‐GCaMP6 mice. [file GLIA-74-0-s012.tif]

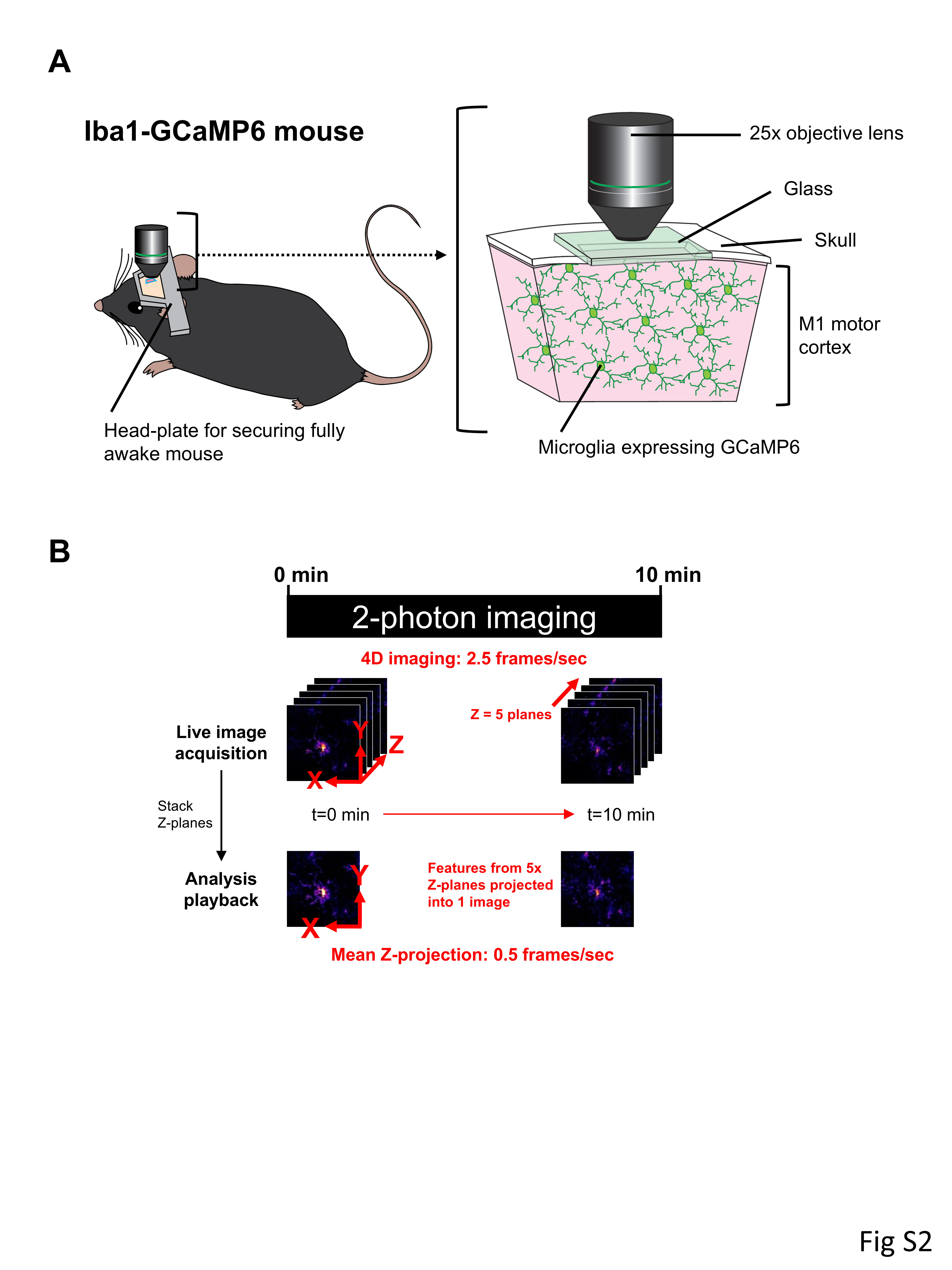

Supplement: Supplementary file 3 — Figure S2: Schematics of the setup and procedure for in vivo two‐photon imaging of microglia. [file GLIA-74-0-s004.tif]

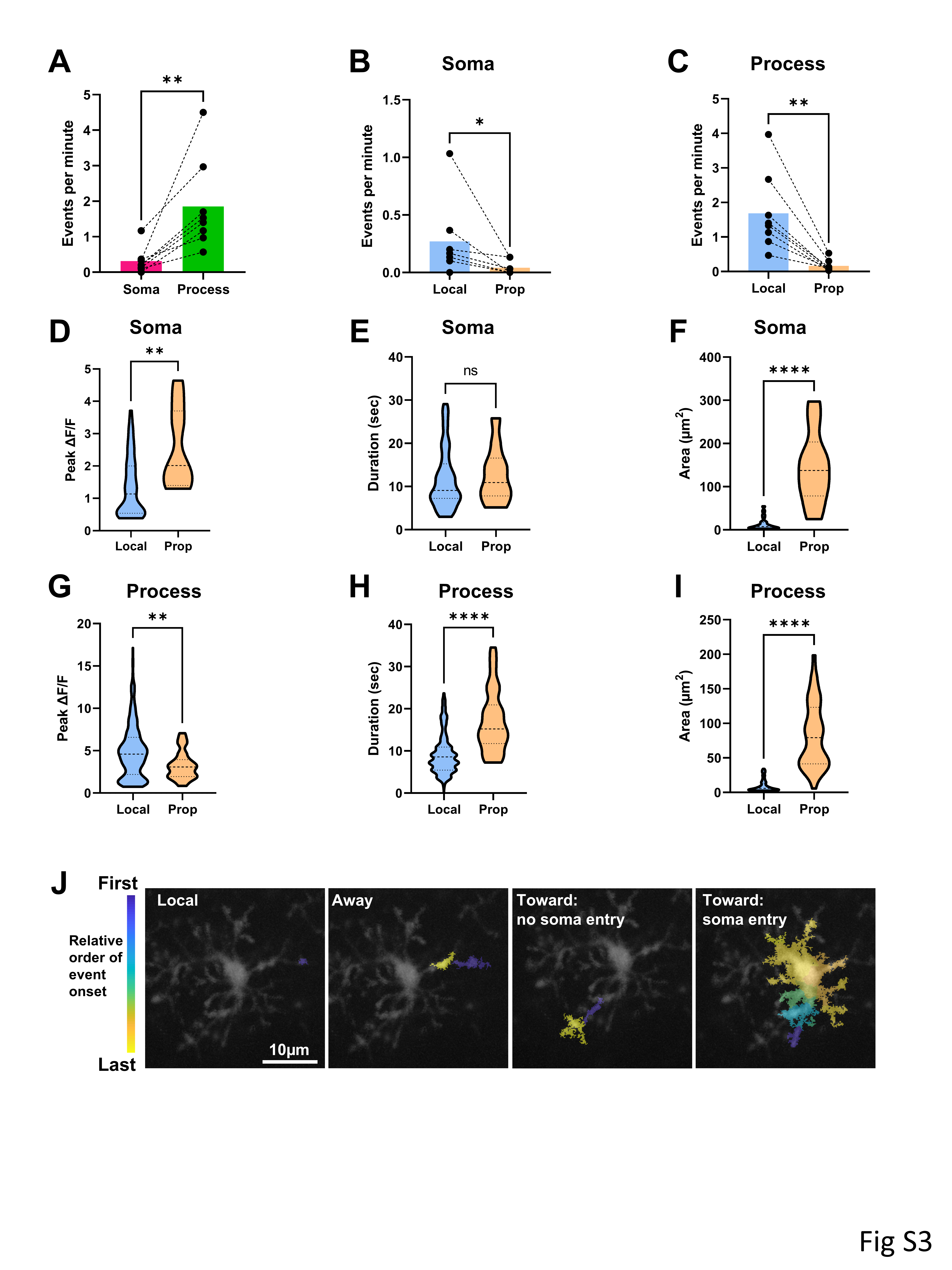

Supplement: Supplementary file 4 — Figure S3: Imaging and characterization of microglial Ca2+ in mice several weeks following cranial window surgery (chronic‐state). [file GLIA-74-0-s009.tif]

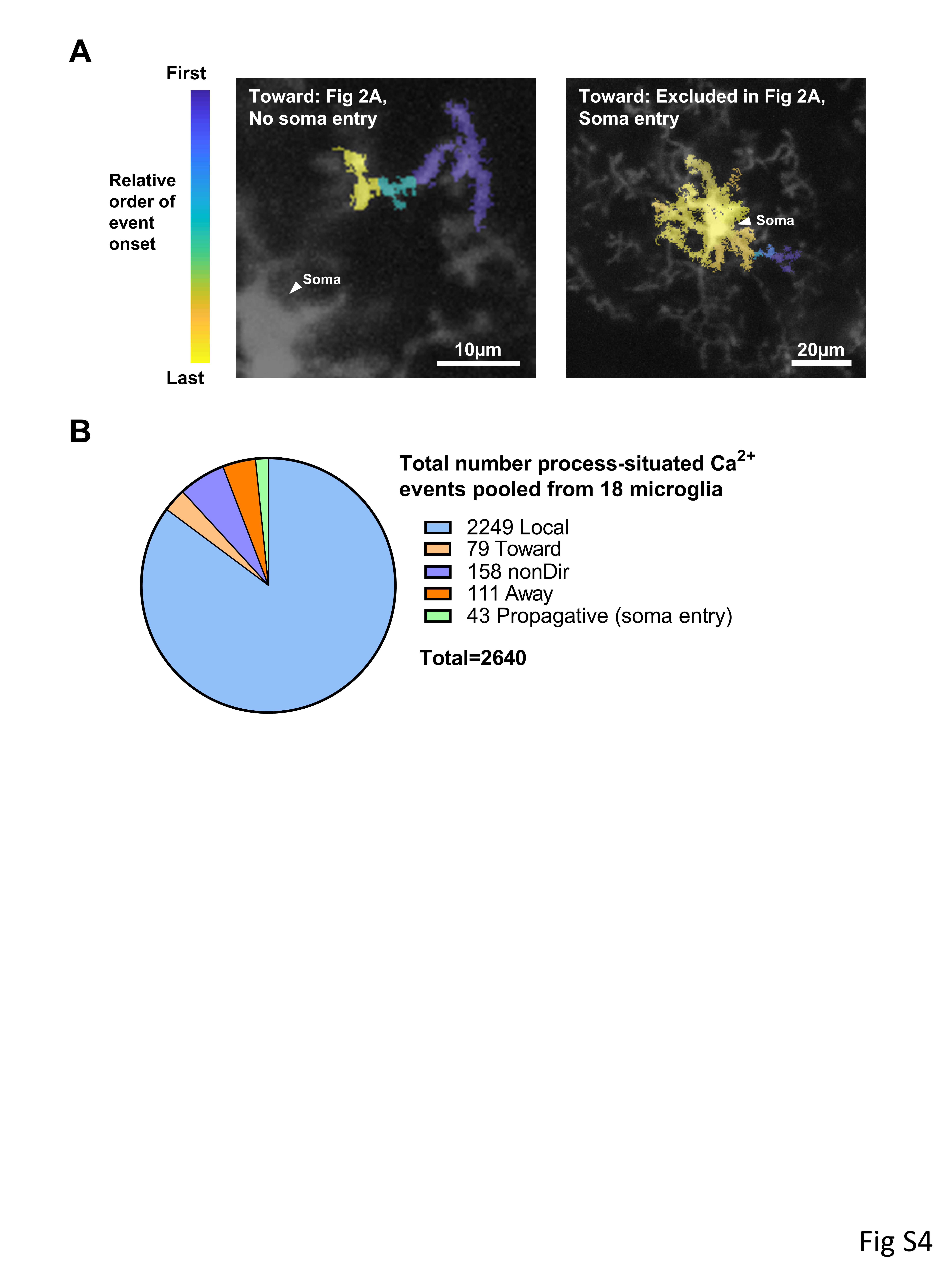

Supplement: Supplementary file 5 — Figure S4: Exclusion of process‐situated “Toward” category Ca2+ events from analysis. [file GLIA-74-0-s008.tif]

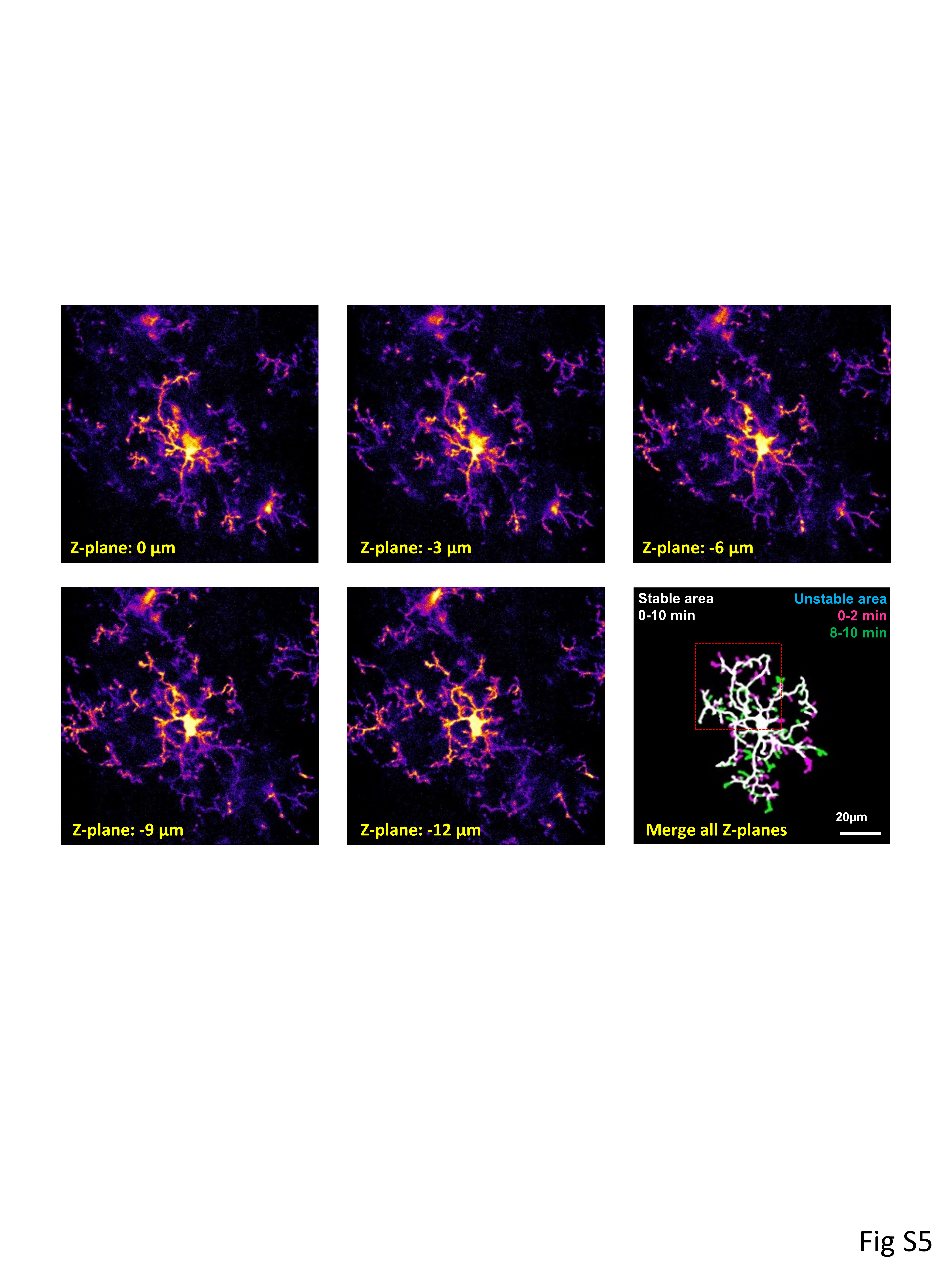

Supplement: Supplementary file 6 — Figure S5: Reconstruction process of the 2‐dimensional microglial morphology map used in Figure 3A,B. [file GLIA-74-0-s003.tif]

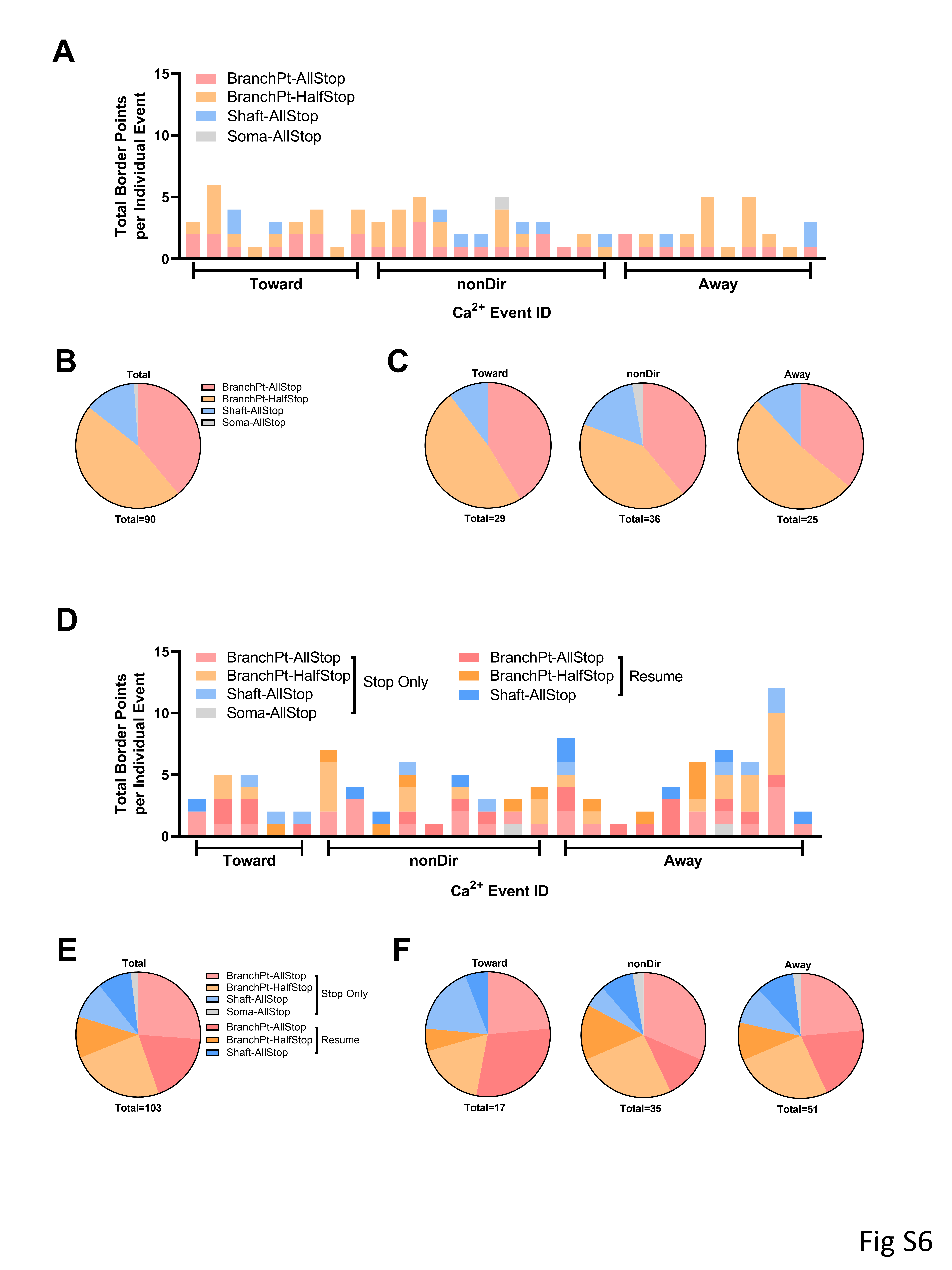

Supplement: Supplementary file 7 — Figure S6: Further characterization of STOP‐GO behavior by propagative process‐situated Ca2+ events. [file GLIA-74-0-s006.tif]

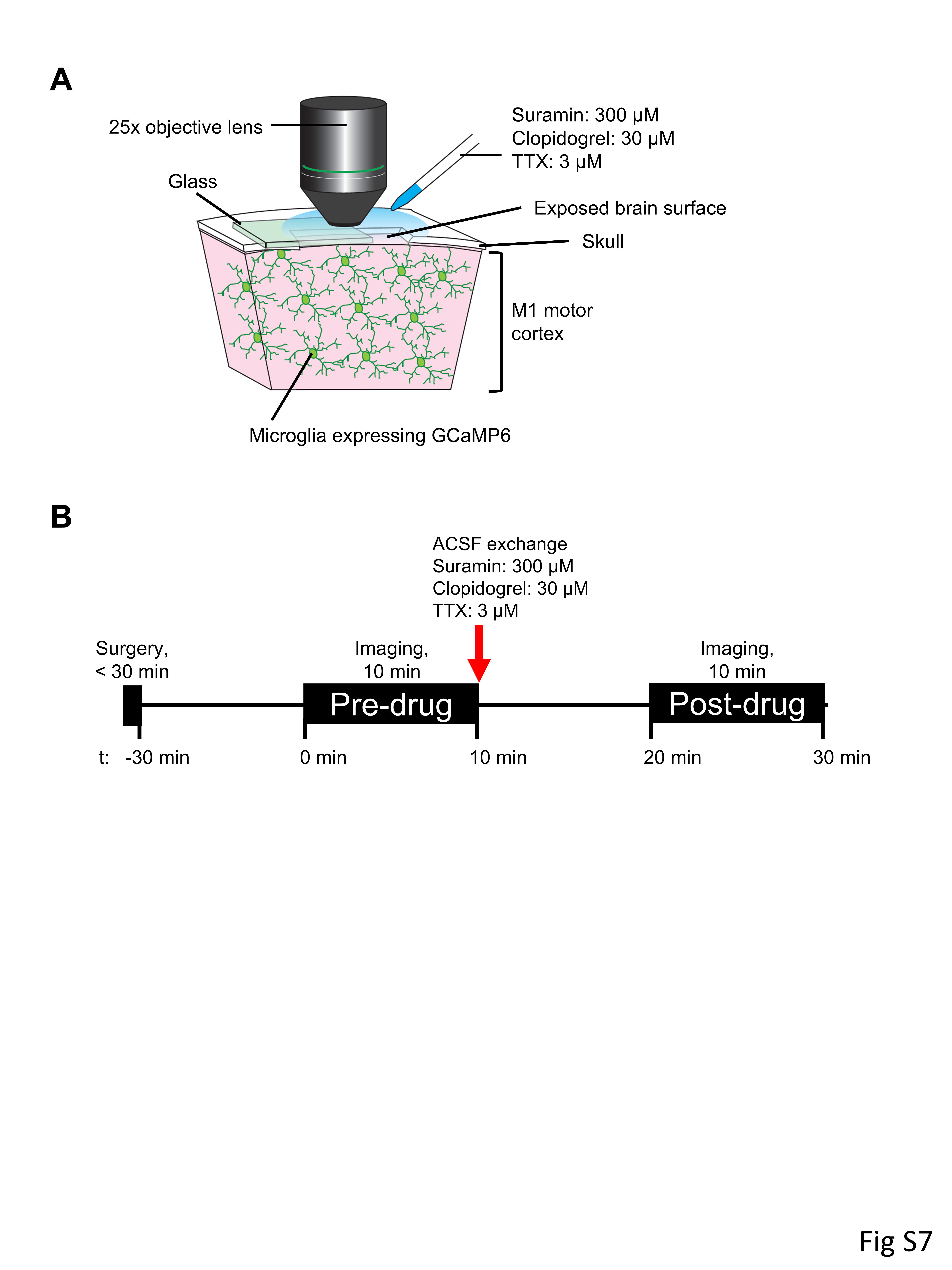

Supplement: Supplementary file 8 — Figure S7: Schematics of the setup and procedure for in vivo two‐photon imaging of microglia. [file GLIA-74-0-s001.tif]

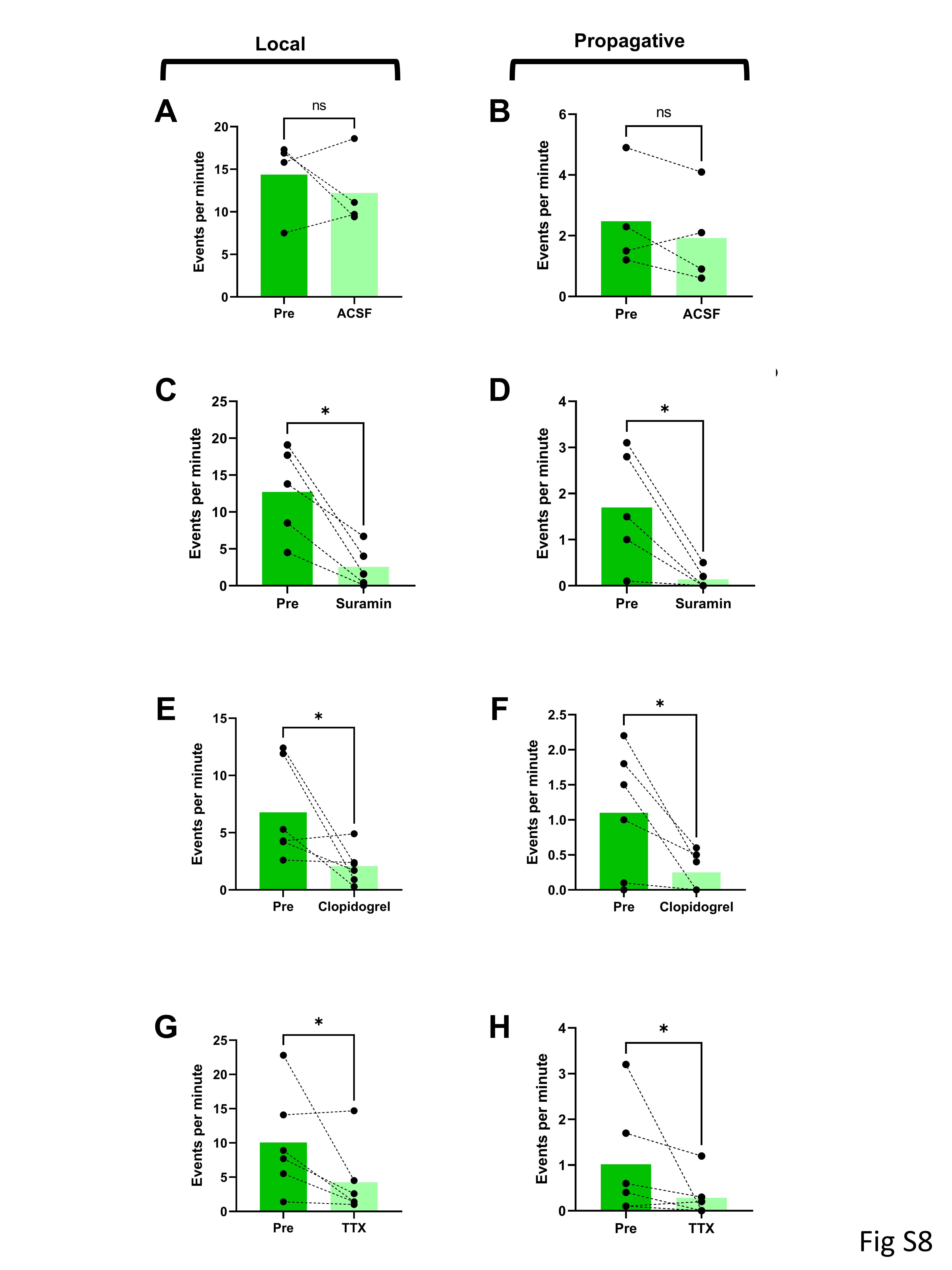

Supplement: Supplementary file 9 — Figure S8: Sub‐categorization of process‐situated microglial Ca2+ events observed in Figure 4 pharmacology experiments as localized vs. propagative. [file GLIA-74-0-s011.tif]

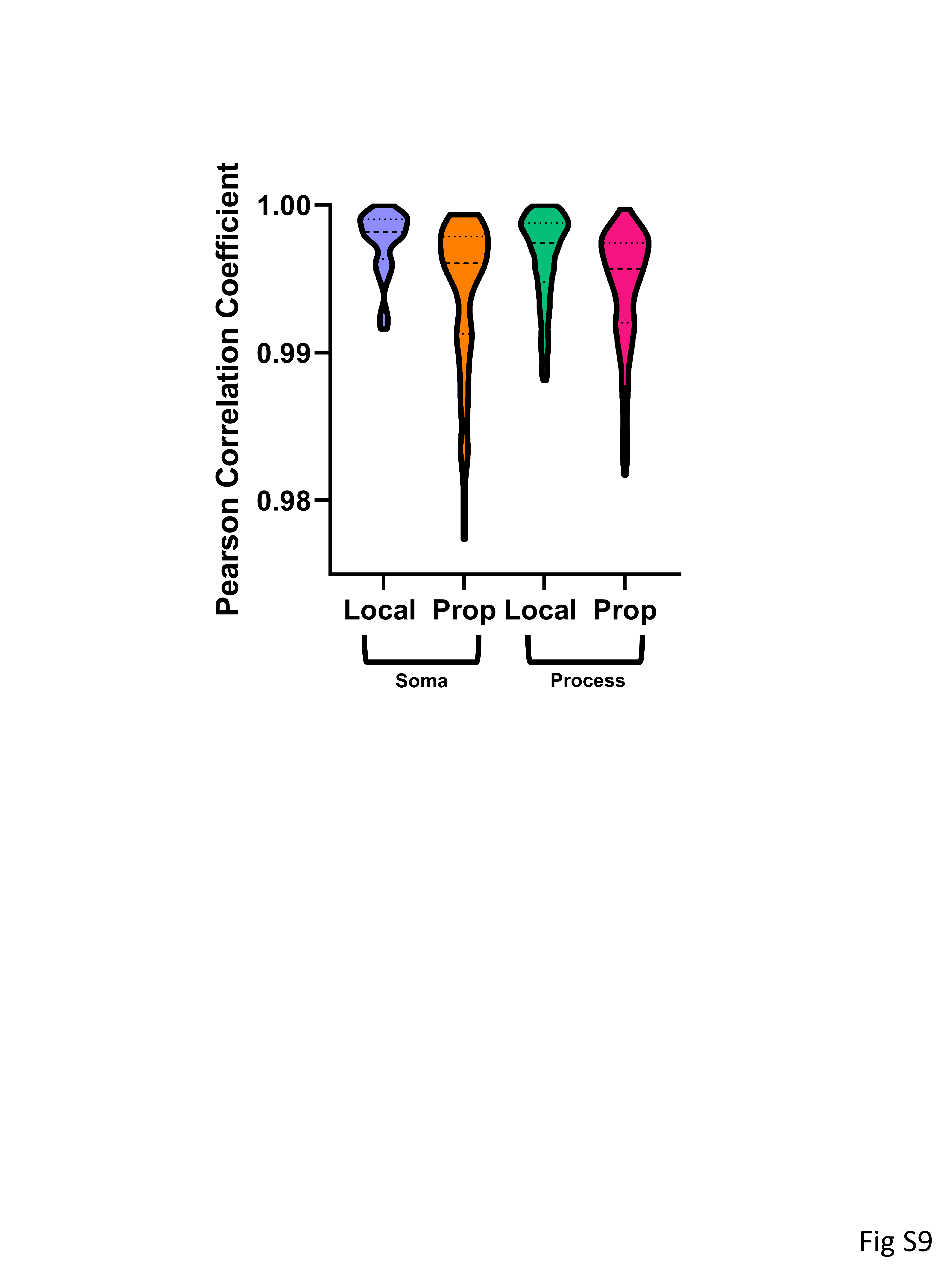

Supplement: Supplementary file 10 — Figure S9: Validation of microglial Ca2+ event detection by the AQuA analysis pipeline. [file GLIA-74-0-s007.tif]
