# Supplementary material for: Spatio‐Temporal Diversity of Calcium Activity in Microglia
Source: Glia. 2026 Jan 6;74(3):e70131. doi: 10.1002/glia.70131 (PMC12772526; doi:10.1002/glia.70131)
Supplement: Supplementary file 2 — Data S1: Supporting information. [file GLIA-74-0-s005.docx]

**Spatio-temporal diversity of calcium activity in microglia**

Hiroshi Horiuchi^1,2,3^, Dennis Lawrence Cheung^1^, Junko Ishida^1,3^, Junichi Nabekura^1,2,4*^

^1^ Division of Homeostatic Development, National Institute for Physiological Science, National Institutes of Natural Sciences, Okazaki, Japan 444-8585

^2^ Department of Physiological Sciences, SOKENDAI: The Graduate University for Advanced Studies, Hayama, Japan 240-0193

^3^ Institute for Research on Next-generation Semiconductor and Sensing Science (IRES²), Toyohashi University of Technology, Toyohashi, Japan 441-8580

^4^ Department of Developmental and Regenerative Neurobiology, Nagoya City University Graduate School of Medical Sciences, Nagoya, Japan 467-0001

**^＊^Correspondence:** nabekura@nips.ac.jp

| **Page** | **Item** |
| --- | --- |
| 2 | Statistics Summary: Fig. 1D-1F |
| 3 | Statistics Summary: Fig. 1G-1H |
| 4 | Statistics Summary: Fig. 1I-1K |
| 5 | Statistics Summary: Fig. 1L-1N |
| 6 | Statistics Summary: Fig. 2B |
| 7 | Statistics Summary: Fig. 2C-2E |
| 8 | Statistics Summary: Fig. 2F-2G |
| 9 | Statistics Summary: Fig. 3C |
| 10 | Statistics Summary: Fig. 4B-4C |
| 11 | Statistics Summary: Fig. 4E-4F, 4H-4I |
| 12 | Statistics Summary: Fig. 4K-4L |
| 13 | Supplementary Figure 1 Description |
| 14 | Supplementary Figure 2 Description |
| 15 | Supplementary Figure 3 Description |
| 18 | Supplementary Figure 4 Description |
| 19 | Supplementary Figure 5 Description |
| 20 | Supplementary Figure 6 Description |
| 21 | Supplementary Figure 7 Description |
| 22 | Supplementary Figure 8 Description |
| 25 | Supplementary Figure 9 Description |

| **1D-1F:**  **Frequency of microglial Ca2+ events as categorized by onset location and propagation propensity** | | | | | | |
| --- | --- | --- | --- | --- | --- | --- |
| ***Wilcoxon matched-pairs sign rank test*** | **1D**  **All Ca^2+^event frequency per cell** | | **1E**  **Soma Ca^2+^ event frequency per cell** | | **1F**  **Process Ca^2+^ event frequency per cell** | |
| **p-value** | <0.0001 | | 0.8073 | | <0.0001 | |
| **significance** | **** | | ns | | **** | |
| **comparison** | Soma vs Process | | Local vs Prop | | | |
| **1-tailed or 2-tailed** | 2-tailed | | | | | |
|  | | | | | | |
| ***Sample size*** | **Soma** | **Process** | **Local** | **Prop** | **Local** | **Prop** |
| **n, cells** | 18 | | | | | |
| **outliers removed prior** | not attempted | | | | | |
| **n, mice** | 15 | | | | | |
|  | | | | | | |
| ***Descriptive statistics*** | ***(events per minute)*** | | | | | |
|  | **Soma** | **Process** | **Local** | **Prop** | **Local** | **Prop** |
| **minimum** | 0.3000 | 4.600 | 0.1000 | 0.1000 | 4.500 | 0.1000 |
| **25% percentile** | 0.4750 | 9.375 | 0.1750 | 0.2750 | 8.250 | 1.075 |
| **median** | 0.7000 | 13.65 | 0.3000 | 0.5000 | 11.10 | 1.900 |
| **75% percentile** | 1.275 | 20.53 | 0.6750 | 0.6000 | 17.40 | 3.125 |
| **maximum** | 1.800 | 24.20 | 1.200 | 0.8000 | 20.30 | 5.500 |
| **range** | 1.500 | 19.60 | 1.100 | 0.7000 | 15.80 | 5.400 |
| **mean** | 0.8944 | 14.67 | 0.4389 | 0.4556 | 12.49 | 2.172 |
| **std. deviation** | 0.4964 | 6.029 | 0.3500 | 0.2093 | 4.952 | 1.508 |
| **std. error of mean** | 0.1170 | 1.421 | 0.08250 | 0.04932 | 1.167 | 0.3554 |

| **1G-1H:**  **AUC’s of microglial Ca^2+^ event trace profiles** | | | | |
| --- | --- | --- | --- | --- |
| ***Mann-Whitney test*** | **1G**  **Soma Ca^2+^ event AUC** | | **1H**  **Process Ca^2+^ event AUC** | |
| **p-value** | <0.0001 | | <0.0001 | |
| **significance** | **** | | **** | |
| **comparison** | Local vs Prop | | | |
| **1-tailed or 2-tailed** | 2-tailed | | | |
|  | | | | |
| ***Sample size*** | **Local** | **Prop** | **Local** | **Prop** |
| **n, events** | 73 | 72 | 2005 | 347 |
| **outliers removed prior** | 6 of 79 | 10 of 82 | 244 of 2249 | 44 of 391 |
| **outlier criteria** | ROUT 1% | | | |
| **n, cells** | 18 (same as 1D) | | | |
| **n, mice** | 15 (same as 1D) | | | |
|  | | | | |
| ***Descriptive statistics*** | ***(ΔF/F.sec)*** | | | |
|  | **Local** | **Prop** | **Local** | **Prop** |
| **minimum** | 2.023 | 5.617 | 0.000 | 8.310 |
| **25% percentile** | 7.412 | 16.84 | 14.26 | 28.17 |
| **median** | 13.21 | 29.07 | 24.03 | 44.80 |
| **75% percentile** | 22.64 | 50.52 | 38.18 | 69.90 |
| **maximum** | 48.45 | 111.2 | 85.12 | 157.0 |
| **range** | 46.42 | 105.6 | 85.12 | 148.7 |
| **mean** | 15.63 | 36.12 | 28.21 | 53.18 |
| **std. deviation** | 10.23 | 25.50 | 18.29 | 33.42 |
| **std. error of mean** | 1.198 | 3.005 | 0.4085 | 1.794 |

| **1I-1K:**  **Temporal and spatial characteristics of soma-situated microglial Ca^2+^ events** | | | | | | |
| --- | --- | --- | --- | --- | --- | --- |
| ***Mann-Whitney test*** | **1I**  **Soma Ca^2+^ event peak amplitude** | | **1J**  **Soma Ca^2+^ event duration** | | **1K**  **Soma Ca^2+^ event area** | |
| **p-value** | 0.0327 | | <0.0001 | | <0.0001 | |
| **significance** | * | | **** | | **** | |
| **comparison** | Local vs Prop | | | | | |
| **1-tailed or 2-tailed** | 2-tailed | | | | | |
|  | | | | | | |
| ***Sample size*** | **Local** | **Prop** | **Local** | **Prop** | **Local** | **Prop** |
| **n, events** | 79 | 82 | 79 | 80 | 67 | 73 |
| **outliers removed prior** | 0 of 79 | 0 of 82 | 0 of 79 | 2 of 82 | 12 of 79 | 9 of 82 |
| **outlier criteria** | ROUT 1% | | | | | |
| **n, cells** | 18 (same as 1D) | | | | | |
| **n, mice** | 15 (same as 1D) | | | | | |
|  | | | | | | |
| ***Descriptive statistics*** | ***(ΔF/F)*** | | ***(sec)*** | | ***(µm^2^)*** | |
|  | **Local** | **Prop** | **Local** | **Prop** | **Local** | **Prop** |
| **minimum** | 0.5752 | 0.6321 | 3.429 | 13.71 | 2.625 | 11.81 |
| **25% percentile** | 1.227 | 1.479 | 12.00 | 20.57 | 4.750 | 54.59 |
| **median** | 1.764 | 1.948 | 17.14 | 27.43 | 8.688 | 106.1 |
| **75% percentile** | 2.216 | 2.908 | 24.00 | 42.00 | 24.44 | 184.6 |
| **maximum** | 4.397 | 5.065 | 54.36 | 75.43 | 60.94 | 401.6 |
| **range** | 3.822 | 4.433 | 50.93 | 61.71 | 58.31 | 389.8 |
| **mean** | 1.830 | 2.189 | 19.22 | 32.44 | 16.05 | 126.1 |
| **std. deviation** | 0.7850 | 1.003 | 11.23 | 15.10 | 15.75 | 88.29 |
| **std. error of mean** | 0.08832 | 0.1107 | 1.264 | 1.688 | 1.924 | 10.33 |

| **1L-1N:**  **Temporal and spatial characteristics of process-situated microglial Ca^2+^ events** | | | | | | |
| --- | --- | --- | --- | --- | --- | --- |
| ***Mann-Whitney test*** | **1L**  **Process Ca^2+^ event peak amplitude** | | **1M**  **Process Ca^2+^ event duration** | | **1N**  **Process Ca^2+^ event area** | |
| **p-value** | 0.1206 | | <0.0001 | | <0.0001 | |
| **significance** | ns | | **** | | **** | |
| **comparison** | Local vs Prop | | | | | |
| **1-tailed or 2-tailed** | 2-tailed | | | | | |
|  | | | | | | |
| ***Sample size*** | **Local** | **Prop** | **Local** | **Prop** | **Local** | **Prop** |
| **n, events** | 2124 | 365 | 2184 | 386 | 1946 | 335 |
| **outliers removed prior** | 125 of 2249 | 26 of 391 | 65 of 2249 | 5 of 391 | 303 of 2249 | 56 of 391 |
| **outlier criteria** | ROUT 1% | | | | | |
| **n, cells** | 18 (same as 1D) | | | | | |
| **n, mice** | 15 (same as 1D) | | | | | |
|  | | | | | | |
| ***Descriptive statistics*** | ***(ΔF/F)*** | | ***(sec)*** | | ***(µm^2^)*** | |
|  | **Local** | **Prop** | **Local** | **Prop** | **Local** | **Prop** |
| **minimum** | 0.6064 | 1.206 | 0.000 | 6.040 | 2.563 | 5.125 |
| **25% percentile** | 2.665 | 2.510 | 8.571 | 18.86 | 3.313 | 10.06 |
| **median** | 3.404 | 3.299 | 14.09 | 25.94 | 4.688 | 18.50 |
| **75% percentile** | 4.418 | 4.391 | 22.29 | 36.06 | 6.813 | 33.13 |
| **maximum** | 8.112 | 8.454 | 48.32 | 73.75 | 12.88 | 74.88 |
| **range** | 7.506 | 7.249 | 48.32 | 67.71 | 10.31 | 69.75 |
| **mean** | 3.657 | 3.575 | 16.47 | 28.86 | 5.400 | 23.55 |
| **std. deviation** | 1.388 | 1.493 | 9.855 | 12.97 | 2.544 | 16.72 |
| **std. error of mean** | 0.03012 | 0.07816 | 0.2109 | 0.6600 | 0.05766 | 0.9135 |

| **2B:**  **Frequency of process-situated microglial Ca^2+^ events as categorized by propagation propensity and directionality** | | | | |
| --- | --- | --- | --- | --- |
| ***Friedman test*** | **p-value** | | 0.1292 | |
|  | **significance** | | ns | |
|  | | | | |
| ***Post hoc Dunn’s multiple comparisons test*** | **adjusted p-value** | | **significance** | |
| **nonDir vs Toward** | >0.9999 | | n/a | |
| **nonDir vs Away** | 0.8360 | | n/a | |
| **Toward vs Away** | 0.1658 | | n/a | |
|  | | | | |
| ***Sample size*** | **nonDir** | **Toward** | | **Away** |
| **n, cells** | 18 (same as 1D) | | | |
| **outliers removed prior** | not attempted | | | |
| **n, mice** | 15 (same as 1D) | | | |
|  | | | | |
| ***Descriptive statistics*** | ***(events per minute)*** | | | |
|  | **Local** | **Toward** | | **Away** |
| **Minimum** | 0.000 | 0.000 | | 0.1000 |
| **25% Percentile** | 0.3500 | 0.2000 | | 0.2000 |
| **Median** | 0.6000 | 0.4000 | | 0.6000 |
| **75% Percentile** | 0.8250 | 0.7500 | | 1.325 |
| **Maximum** | 1.700 | 1.700 | | 1.700 |
| **Range** | 1.700 | 1.700 | | 1.600 |
| **Mean** | 0.6333 | 0.5389 | | 0.7611 |
| **Std. Deviation** | 0.4339 | 0.5043 | | 0.5543 |
| **Std. Error of Mean** | 0.1023 | 0.1189 | | 0.1306 |

| **2C-2E:**  **Temporal and spatial characteristics of process-situated microglial Ca^2+^ events as categorized by propagation propensity and directionality** | | | | | | | | | | | | |
| --- | --- | --- | --- | --- | --- | --- | --- | --- | --- | --- | --- | --- |
| ***Kruskall-Wallis test*** | **2C**  **Process Ca^2+^ event peak amplitude** | | | | **2D**  **Process Ca^2+^ event duration** | | | | **2E**  **Process Ca^2+^ event area** | | | |
| **p-value** | 0.4465 | | | | 0.7396 | | | | 0.1439 | | | |
| **significance** | ns | | | | ns | | | | ns | | | |
|  | | | | | | | | | | | | |
| ***Post hoc Dunn’s multiple comparisons test*** | **adjusted p-value** | | **significance** | | **adjusted p-value** | | **significance** | | **adjusted p-value** | | **significance** | |
| **nonDir vs Toward** | >0.9999 | | n/a | | >0.9999 | | n/a | | >0.9999 | | n/a | |
| **nonDir vs Away** | >0.9999 | | n/a | | >0.9999 | | n/a | | 0.3809 | | n/a | |
| **Toward vs Away** | 0.6323 | | n/a | | >0.9999 | | n/a | | 0.1986 | | n/a | |
|  | | | | | | | | | | | | |
| ***Sample size*** | **nonDir** | **Toward** | | **Away** | **nonDir** | **Toward** | | **Away** | **nonDir** | **Toward** | | **Away** |
| **n, events** | 147 | 75 | | 101 | 157 | 79 | | 110 | 151 | 70 | | 101 |
| **outliers removed prior** | 11 of 158 | 4 of 79 | | 10 of 111 | 1 of 158 | 0 of 79 | | 1 of 111 | 7 of 158 | 9 of 79 | | 10 of 111 |
| **outlier criteria** | ROUT 1% | | | | | | | | | | | |
| **n, cells** | 18 (same as 1D) | | | | | | | | | | | |
| **n, mice** | 15 (same as 1D) | | | | | | | | | | | |
|  | | | | | | | | | | | | |
| ***Descriptive statistics*** | ***(ΔF/F)*** | | | | ***(sec)*** | | | | ***(µm^2^)*** | | | |
|  | **nonDir** | **Toward** | | **Away** | **nonDir** | **Toward** | | **Away** | **nonDir** | **Toward** | | **Away** |
| **minimum** | 1.399 | 1.522 | | 1.293 | 6.857 | 6.040 | | 8.571 | 5.125 | 5.563 | | 5.250 |
| **25% percentile** | 2.584 | 2.650 | | 2.521 | 20.03 | 18.86 | | 18.67 | 10.00 | 9.500 | | 11.75 |
| **median** | 3.494 | 3.501 | | 3.225 | 25.71 | 26.17 | | 26.17 | 18.69 | 15.50 | | 19.13 |
| **75% percentile** | 4.417 | 4.670 | | 4.521 | 34.29 | 37.71 | | 39.54 | 31.25 | 32.70 | | 39.06 |
| **maximum** | 8.198 | 7.605 | | 8.807 | 66.86 | 60.40 | | 73.75 | 65.00 | 58.69 | | 80.25 |
| **range** | 6.799 | 6.083 | | 7.514 | 60.00 | 54.36 | | 65.18 | 59.88 | 53.13 | | 75.00 |
| **mean** | 3.708 | 3.778 | | 3.651 | 27.81 | 28.93 | | 29.87 | 22.30 | 21.06 | | 26.77 |
| **std. deviation** | 1.504 | 1.361 | | 1.673 | 12.19 | 13.36 | | 14.40 | 14.87 | 14.57 | | 19.39 |
| **std. error of mean** | 0.1240 | 0.1571 | | 0.1665 | 0.9728 | 1.503 | | 1.373 | 1.210 | 1.742 | | 1.929 |

| **2F-2G**  **Spatiotemporal characteristics of process-situated microglial Ca^2+^ events as categorized by propagation directionality** | | | | |
| --- | --- | --- | --- | --- |
| ***Mann-Whitney test*** | **2F**  **Process Ca^2+^ event propagation speed** | | **2G**  **Process Ca^2+^ event expansion rate** | |
| **p-value** | 0.2002 | | 0.8784 | |
| **significance** | ns | | ns | |
| **comparison** | Toward vs Away | | | |
| **1-tailed or 2-tailed** | 2-tailed | | | |
|  | | | | |
| ***Sample size*** | **Toward** | **Away** | **Toward** | **Away** |
| **n, events** | 71 | 106 | 72 | 97 |
| **outliers removed prior** | 8 of 79 | 5 of 111 | 7 of 79 | 14 of 111 |
| **outlier criteria** | ROUT 1% | | | |
| **n, cells** | 18 (same as 1D) | | | |
| **n, mice** | 15 (same as 1D) | | | |
|  | | | | |
| ***Descriptive statistics*** | ***(µm/sec)*** | | ***(µm^2^/sec)*** | |
|  | **Toward** | **Away** | **Toward** | **Away** |
| **minimum** | 0.01552 | 0.01493 | 0.09042 | 0.1604 |
| **25% percentile** | 0.07988 | 0.07980 | 0.3931 | 0.4021 |
| **median** | 0.1145 | 0.1331 | 0.6727 | 0.7656 |
| **75% percentile** | 0.1902 | 0.2182 | 1.260 | 1.132 |
| **maximum** | 0.3750 | 0.4545 | 2.563 | 2.301 |
| **range** | 0.3595 | 0.4396 | 2.473 | 2.141 |
| **mean** | 0.1391 | 0.1628 | 0.8632 | 0.8309 |
| **std. deviation** | 0.08506 | 0.1051 | 0.6222 | 0.5092 |
| **std. error of mean** | 0.01009 | 0.01021 | 0.07333 | 0.05171 |

| **3C:**  **Probability of Ca^2+^ compartmentalization occurring at microglial branch points** | | |
| --- | --- | --- |
| ***Binomial exact test*** | **p-value** | <0.0001 |
|  | **significance** | **** |
|  | **null hypothesis, H_0_** | 50% chance |
|  | | |
| ***Sample size*** | **Microglia branch point restricts Ca^2+^ spread** | **Ca^2+^ spread not restricted** |
| **n, Ca^2+^ events** | 55/55 | 0/55 |
|  | 33/55 (BranchPt-FullStop & BranchPt-HalfStop)  15/55 (BranchPt-FullStop only)  7/55 (BranchPt-HalfStop only) |  |
| **n, cells** | 18 (same as 1D) | |
| **n, mice** | 15 (same as 1D) | |

| **4B-4C:**  **Drug application procedure (control, ACSF) and microglial Ca^2+^ activity** | | | | |
| --- | --- | --- | --- | --- |
| ***Wilcoxon matched-pairs sign rank test*** | **4B**  **Soma Ca^2+^ event frequency** | | **4C**  **Process Ca^2+^ event frequency** | |
| **p-value** | 0.3750 | | 0.3125 | |
| **significance** | ns | | ns | |
| **comparison** | Pre vs ACSF | | | |
| **1-tailed or 2-tailed** | 1-tailed | | | |
|  | | | | |
| ***Sample size*** | **Pre** | **ACSF** | **Pre** | **ACSF** |
| **n, cells** | 4 | | | |
| **outliers removed prior** | not attempted | | | |
| **n, mice** | 3 | | | |
|  | | | | |
| ***Descriptive statistics*** | ***(events per minute)*** | | | |
|  | **Pre** | **ACSF** | **Pre** | **ACSF** |
| **minimum** | 0.7000 | 0.8000 | 9.000 | 10.00 |
| **25% percentile** | 0.7750 | 0.8250 | 11.38 | 10.45 |
| **median** | 1.050 | 1.050 | 18.85 | 11.90 |
| **75% percentile** | 1.400 | 1.500 | 20.33 | 20.03 |
| **maximum** | 1.500 | 1.600 | 20.70 | 22.70 |
| **range** | 0.8000 | 0.8000 | 11.70 | 12.70 |
| **mean** | 1.075 | 1.125 | 16.85 | 14.13 |
| **std. deviation** | 0.3304 | 0.3594 | 5.313 | 5.787 |
| **std. error of mean** | 0.1652 | 0.1797 | 2.657 | 2.893 |

| **4E-4F, 4H-4I:**  **Purinergic signalling (P2Y antagonists) and microglial Ca^2+^ activity** | | | | | | | | |
| --- | --- | --- | --- | --- | --- | --- | --- | --- |
| ***Wilcoxon matched-pairs sign rank test*** | **Suramin (300 µM)** | | | | **Clopidogrel (30 µM)** | | | |
|  | **4H**  **Soma** | | **4I**  **Process** | | **4K**  **Soma** | | **4L**  **Process** | |
| **p-value** | 0.0313 | | 0.0313 | | 0.2031 | | 0.0313 | |
| **significance** | * | | * | | ns | | * | |
| **comparison** | Pre vs Drug | | | | | | | |
| **1-tailed or 2-tailed** | 1-tailed | | | | | | | |
|  | | | | | | | | |
| ***Sample size*** | **Pre** | **Suramin** | **Pre** | **Suramin** | **Pre** | **Clopidogrel** | **Pre** | **Clopidogrel** |
| **n, cells** | 5 | | | | 6 | | | |
| **outliers removed prior** | not attempted | | | | | | | |
| **n, mice** | 4 | | | | 5 | | | |
|  | | | | | | | | |
| ***Descriptive statistics*** | ***(events per minute)*** | | | | | | | |
|  | **Pre** | **Suramin** | **Pre** | **Suramin** | **Pre** | **Clopidogrel** | **Pre** | **Clopidogrel** |
| **Minimum** | 0.3000 | 0.000 | 4.600 | 0.1000 | 0.000 | 0.000 | 2.700 | 0.3000 |
| **25% Percentile** | 0.5000 | 0.000 | 7.050 | 0.2500 | 0.2250 | 0.1500 | 4.650 | 0.7500 |
| **Median** | 0.7000 | 0.000 | 16.90 | 1.800 | 1.100 | 0.2000 | 5.650 | 2.350 |
| **75% Percentile** | 1.400 | 0.6500 | 20.55 | 5.600 | 1.425 | 0.8500 | 13.70 | 3.375 |
| **Maximum** | 1.800 | 1.200 | 20.60 | 7.200 | 1.500 | 1.900 | 14.60 | 5.400 |
| **Range** | 1.500 | 1.200 | 16.00 | 7.100 | 1.500 | 1.900 | 11.90 | 5.100 |
| **Mean** | 0.9000 | 0.2600 | 14.42 | 2.700 | 0.9000 | 0.5000 | 7.883 | 2.333 |
| **Std. Deviation** | 0.5612 | 0.5273 | 7.105 | 2.950 | 0.6229 | 0.7043 | 4.885 | 1.774 |
| **Std. Error of Mean** | 0.2510 | 0.2358 | 3.178 | 1.319 | 0.2543 | 0.2875 | 1.994 | 0.7242 |

| **4K-4L:**  **TTX (3 µM) application and microglial Ca^2+^ activity** | | | | |
| --- | --- | --- | --- | --- |
| ***Wilcoxon matched-pairs sign rank test*** | **4K**  **Soma Ca^2+^ event frequency** | | **4L**  **Process Ca^2+^ event frequency** | |
| **p-value** | 0.0625 | | 0.0313 | |
| **significance** | ns | | * | |
| **comparison** | Pre vs Drug | | | |
| **1-tailed or 2-tailed** | 1-tailed | | | |
|  | | | | |
| ***Sample size*** | **Pre** | **TTX** | **Pre** | **TTX** |
| **n, cells** | 6 | | | |
| **outliers removed prior** | not attempted | | | |
| **n, mice** | 6 | | | |
|  | | | | |
| ***Descriptive statistics*** | ***(events per minute)*** | | | |
|  | **Pre** | **TTX** | **Pre** | **TTX** |
| **minimum** | 0.000 | 0.000 | 1.500 | 1.200 |
| **25% percentile** | 0.2250 | 0.2250 | 4.950 | 1.350 |
| **median** | 1.550 | 0.4000 | 8.550 | 2.150 |
| **75% percentile** | 1.925 | 0.9000 | 18.35 | 7.350 |
| **maximum** | 2.000 | 0.9000 | 26.00 | 15.90 |
| **range** | 2.000 | 0.9000 | 24.50 | 14.70 |
| **mean** | 1.217 | 0.4833 | 11.08 | 4.550 |
| **std. deviation** | 0.8658 | 0.3601 | 8.656 | 5.690 |
| **std. error of mean** | 0.3535 | 0.1470 | 3.534 | 2.323 |

**Supplementary Figure 1**

**GCaMP6 is exclusively expressed by microglia in transgenic Iba1-GCaMP6 mice.**

**A.** In Iba1-GCaMP6 mice, the tetO-tTA conditional expression system couples GCaMP6 expression to the ionized Ca^2+^ binding adapter molecule 1 (Iba1) promoter. The Iba1 promoter is only active in microglia and drives expression of the tetracycline transactivator (tTA) gene. The tetracycline response element (tetO) promoter becomes activated when bound by tTA and drives expression of the GCaMP6 gene. This gene expression setup can be reversibly disabled by doxycycline (DOX) as DOX-tTA binding prevents tTA-tetO binding. Thus, GCaMP6 is exclusively expressed in microglia and can be reversibly switched on and off.

**B.** Immunohistochemistry confirms exclusive expression of GCaMP6 (green) in Iba1-positive (red) microglia. Surprisingly, not all Iba1-positive microglia co-express GCaMP6. Scale bar = 50 µm.

**Supplementary Figure 2**

**Schematics of the setup and procedure for *in vivo* 2-photon imaging of microglia.**

**A.** *In vivo* 2-photon imaging of microglia was performed in fully awake mice. The custom-made metal head plate enabled the mouse’s head to be secured and immobilized under the 25x microscope objective lens. For imaging-only experiments, the cranial window was prepared such that the double-layered glass coverslip fully sealed the craniotomy made over the left motor cortex (M1). Thus, GCaMP6 expressing microglia in M1 motor cortex could be imaged in fully awake mice with minimal motion-induced artefact.

**B.** Four dimensional imaging of microglial Ca^2+^ activity was performed in 10-minute epochs. In all cases, the acquisition rate was 2.5 frames/sec with XY-dimensions of 512 x 512 pixel^2^, 0.25 µm/pixel, and a Z-dimension of 5 Z-planes. The 5 Z-planes were flattened into 1 plane for AQuA-based analysis thus making the effective rate 0.5 frames/sec.

**Supplementary Figure 3**

**Imaging and characterization of microglial Ca^2+^ in mice several weeks following cranial window surgery (chronic-state).**

**A-C.** Dot plots quantifying the frequency of microglial Ca^2+^ events as classified by their location and spreading dynamics. Each pair of data points represents one microglia cell, n = 8. Columns indicate cohort means. **p< 0.01, *p <0.05; Wilcoxon’s matched-pairs sign ranked test, 2-tailed. **(A)** All Ca^2+^ events per microglia: soma-situated (Soma) vs process-situated (Process). **(B)** Soma-situated Ca^2+^ events: localized (Local) vs propagative (Prop). **(C)** Process-situated Ca^2+^ events: localized (Local) vs propagative (Prop).

|  | **Supplementary 3A** | | **Supplementary 3B** | | **Supplementary 3C** | |
| --- | --- | --- | --- | --- | --- | --- |
| ***Wilcoxon matched-pairs sign rank test*** | **All Ca^2+^event frequency per cell** | | **Soma Ca^2+^ event frequency per cell** | | **Process Ca^2+^ event frequency per cell** | |
| **p-value** | 0.0078 | | 0.0156 | | 0.0078 | |
| **significance** | ** | | * | | ** | |
| **comparison** | **Soma vs Process** | | **Local vs Prop** | | | |
| **1-tailed or 2-tailed** | 2-tailed | | | | | |
|  | | | | | | |
| ***Sample size*** | **Soma** | **Process** | **Local** | **Prop** | **Local** | **Prop** |
| **n, cells** | 8 | | | | | |
| **outliers removed prior** | not attempted | | | | | |
| **n, mice** | 7 | | | | | |
|  | | | | | | |
| ***Descriptive statistics*** | ***(events per minute)*** | | | | | |
|  | **Soma** | **Process** | **Local** | **Prop** | **Local** | **Prop** |
| **minimum** | 0.000 | 0.5667 | 0.000 | 0.000 | 0.4667 | 0.03333 |
| **25% percentile** | 0.1083 | 1.017 | 0.1083 | 0.000 | 0.9333 | 0.06667 |
| **median** | 0.2000 | 1.467 | 0.1667 | 0.01667 | 1.367 | 0.1000 |
| **75% percentile** | 0.3583 | 2.650 | 0.3250 | 0.1083 | 2.408 | 0.2583 |
| **maximum** | 1.167 | 4.500 | 1.033 | 0.1333 | 3.967 | 0.5333 |
| **range** | 1.167 | 3.933 | 1.033 | 0.1333 | 3.500 | 0.5000 |
| **mean** | 0.3125 | 1.850 | 0.2708 | 0.04167 | 1.683 | 0.1667 |
| **std. deviation** | 0.3651 | 1.281 | 0.3249 | 0.05842 | 1.123 | 0.1690 |
| **std. error of mean** | 0.1291 | 0.4529 | 0.1149 | 0.02065 | 0.3972 | 0.05976 |

**D-I.** Truncated violin plots quantifying the temporal and spatial characteristics of microglial Ca^2+^ events. ****p< 0.0001, ***p< 0.001, **p< 0.01, *p <0.05; Mann-Whitney test, 2-tailed. Cohorts [localized (Local), propagative (Prop)], ROUT 1% outlier exclusion used. **(D)** Peak amplitude of soma-situated Ca^2+^ events, n = [61, 10]. **(E)** Duration of soma-situated Ca^2+^ events, n = [56, 10]. **(F)** Area of soma-situated Ca^2+^ events, n = [53, 10]. **(G)** Peak amplitude of process-situated Ca^2+^ events, n = [404, 39]. **(H)** Duration of process-situated Ca^2+^ events, n = [365, 38]. **(I)** Area of process-situated Ca^2+^ events, n = [341, 39].

|  | **Supplementary 3D** | | **Supplementary 3E** | | **Supplementary 3F** | |
| --- | --- | --- | --- | --- | --- | --- |
| ***Mann-Whitney test*** | **Soma Ca^2+^ event peak amplitude** | | **Soma Ca^2+^ event duration** | | **Soma Ca^2+^ event area** | |
| **p-value** | 0.0024 | | 0.4993 | | <0.0001 | |
| **significance** | ** | | ns | | **** | |
| **comparison** | Local vs Prop | | | | | |
| **1-tailed or 2-tailed** | 2-tailed | | | | | |
|  | | | | | | |
| ***Sample size*** | **Local** | **Prop** | **Local** | **Prop** | **Local** | **Prop** |
| **n, events** | 61 | 10 | 56 | 10 | 53 | 10 |
| **outliers removed prior** | 4 of 65 | 0 of 10 | 9 of 65 | 0 of 10 | 12 of 65 | 0 of 10 |
| **outlier criteria** | ROUT 1% | | | | | |
| **n, cells** | 8 (same as S5A) | | | | | |
| **n, mice** | 7 (same as S5A) | | | | | |
|  | | | | | | |
| ***Descriptive statistics*** | ***(ΔF/F)*** | | ***(sec)*** | | ***(µm^2^)*** | |
|  | **Local** | **Prop** | **Local** | **Prop** | **Local** | **Prop** |
| **minimum** | 0.3855 | 1.296 | 2.990 | 5.158 | 2.563 | 24.75 |
| **25% percentile** | 0.5405 | 1.399 | 7.265 | 7.809 | 3.750 | 78.41 |
| **median** | 1.134 | 2.013 | 9.086 | 10.92 | 7.438 | 137.7 |
| **75% percentile** | 2.001 | 3.701 | 15.24 | 16.60 | 17.22 | 203.5 |
| **maximum** | 3.708 | 4.639 | 29.09 | 25.79 | 54.50 | 297.1 |
| **range** | 3.323 | 3.343 | 26.10 | 20.63 | 51.94 | 272.4 |
| **mean** | 1.352 | 2.534 | 11.70 | 12.59 | 13.04 | 144.5 |
| **std. deviation** | 0.9268 | 1.238 | 6.856 | 6.563 | 13.72 | 84.59 |
| **std. error of mean** | 0.1187 | 0.3916 | 0.9161 | 2.075 | 1.885 | 26.75 |

|  | **Supplementary 3G** | | **Supplementary 3H** | | **Supplementary 3I** | |
| --- | --- | --- | --- | --- | --- | --- |
| ***Mann-Whitney test*** | **Process Ca^2+^ event peak amplitude** | | **Process Ca^2+^ event duration** | | **Process Ca^2+^ event area** | |
| **p-value** | 0.0024 | | <0.0001 | | <0.0001 | |
| **significance** | ** | | **** | | **** | |
| **comparison** | Local vs Prop | | | | | |
| **1-tailed or 2-tailed** | 2-tailed | | | | | |
|  | | | | | | |
| ***Sample size*** | **Local** | **Prop** | **Local** | **Prop** | **Local** | **Prop** |
| **n, events** | 404 | 39 | 365 | 38 | 341 | 39 |
| **outliers removed prior** | 0 of 404 | 1 of 40 | 39 of 404 | 2 of 40 | 63 of 404 | 1 of 40 |
| **outlier criteria** | ROUT 1% | | | | | |
| **n, cells** | 8 (same as S5A) | | | | | |
| **n, mice** | 7 (same as S5A) | | | | | |
|  | | | | | | |
| ***Descriptive statistics*** | ***(ΔF/F)*** | | ***(sec)*** | | ***(µm^2^)*** | |
|  | **Local** | **Prop** | **Local** | **Prop** | **Local** | **Prop** |
| **minimum** | 0.7379 | 0.8339 | 0.000 | 7.265 | 2.563 | 5.813 |
| **25% percentile** | 2.191 | 1.938 | 5.460 | 11.75 | 3.563 | 41.25 |
| **median** | 4.599 | 3.083 | 8.596 | 15.21 | 6.000 | 79.25 |
| **75% percentile** | 6.581 | 3.941 | 10.91 | 20.92 | 13.69 | 123.1 |
| **maximum** | 17.13 | 7.060 | 23.64 | 34.51 | 34.13 | 198.4 |
| **range** | 16.39 | 6.226 | 23.64 | 27.25 | 31.56 | 192.6 |
| **mean** | 4.857 | 3.271 | 9.260 | 17.08 | 9.795 | 82.28 |
| **std. deviation** | 3.083 | 1.632 | 4.688 | 7.347 | 8.649 | 47.28 |
| **std. error of mean** | 0.1534 | 0.2614 | 0.2454 | 1.192 | 0.4684 | 7.570 |

**J.** Representative Ca^2+^ events originating in the same microglial process but exhibiting different spatio-temporal dynamics (colour scale). Ca^2+^ events may remain localized (left-most panel, “Local”), propagate away the soma (middle-left panel, “Away”), propagate toward the soma without entry (middle-right panel, “Toward: no soma entry”), or propagate toward and enter the soma (right-most panel, “Toward: soma entry”). Scale bar = 10 µm, arrowhead indicates microglial soma.

**Supplementary Figure 4**

**Exclusion of process-situated “Toward” category Ca^2+^ events from analysis.**

**A.** In Figure 2 of the main text, process-situated propagative Ca^2+^ events were further categorized based on whether they propagated “Toward” or “Away” from the microglial soma. “Toward” Ca^2+^ events either propagated entirely within the microglial process (left panel) or eventually propagated out of the microglial process and entered into the microglial soma (right panel). The former were included for analysis whereas the latter was excluded. This is because a Ca^2+^ event’s entry into the microglial soma was often followed by its erratic spread into all of the surrounding microglial processes. Thus, such Ca^2+^ could no longer be neatly categorized as propagating predominantly “Toward” or “Away” from the microglial soma. The spatio-temporal dynamics (colour scale) of both sub-categories of “Toward” Ca^2+^ events and the microglial soma (arrowhead) are indicated. Note the differing scale bars, left = 10 µm, right = 20 µm.

**B.** Pie chart summarizing the overall frequencies of the various categories of process-situated microglial Ca^2+^ events.

**Supplementary Figure 5**

**Reconstruction process of the 2-dimensional microglial morphology map used in Figure 3A-3B.**

The top, bottom-left, and bottom-middle panels are mean intensity projections of GCaMP6 fluorescence at different Z-axis depths. These are overlaid to generate the 2-dimensional microglial morphology map in the bottom right panel. Stable regions of the microglia do not move throughout the entire 10-minute imaging period and are indicated in white. Unstable regions of the microglia are only present in either the first or last 2 minutes of the imaging period and are indicated in pink and green, respectively. AQuA-based deconvolution is only possible for Ca^2+^ activity occurring in stable regions. The area demarcated by the red dashed square corresponds to the top-left panels in Figure 3A-3B in the main text. Note that the apparently looped structure is actually 2 separate microglial processes that overlap each other at different Z-axis depths. Scale bar = 20 µm.

**Supplementary Figure 6**

**Further characterization of STOP-GO behaviour by propagative process-situated Ca^2+^ events.**

In Figure 3C-3E, the top ~20% by Δ pixel area of propagative process-situated Ca^2+^ events (n = 55) were analysed for STOP-GO behaviour at microglial branch points. Strikingly, all n =55/55 of these Ca^2+^ events exhibited STOP behaviour at a microglial branch point, either completely (BranchPt-AllStop – no propagation into either daughter branch) or partially bounded (BranchPt-HalfStop – propagation into one daughter branch but exclusion from the other). However, only n = 24/55 of these Ca^2+^ events subsequently resumed their propagation past the original microglial branch point.

The same data from Figure 3C–3E are replotted here in the same format, but separated according to whether the Ca²⁺ event subsequently resumed propagation.

**A-C.** Ca^2+^ events which did not resume propagation, n = 31/55 from 15 mice.

**D-F.** Ca^2+^ events which resumed propagation past the original microglial branch point, n = 24/55 from 15 mice. Note that STOP sites (BranchPt-AllStop, BranchPt-HalfStop, Shaft-AllStop, Soma-AllStop) at which Ca^2+^ propagation subsequently resumed (Resume) have been distinguished from sites where the STOP was permanent (Stop Only). STOP behaviour lasted for at least 2 seconds in all cases at all sites.

**Supplementary Figure 7**

**Schematics of the setup and procedure for *in vivo* 2-photon imaging of microglia.**

**A.** For pharmacology experiments, the cranial window was prepared such that the double-layered glass coverslip did not fully seal the craniotomy made over the left motor cortex (M1), leaving a small gap on one side. Thus, drugs diluted in ACSF could be directly applied to the exposed brain surface through this small gap. Care was taken to ensure the exposed brain surface was always covered by ACSF so as to avoid desiccative injury. The double-layered glass coverslip was necessary to reduce breathing-associated movement of the brain.

**B.** Summary of the experimental workflow for comparing microglial Ca^2+^ activity pre- and post-application of drugs. Note that drug application was performed 10 minutes before starting the post-drug imaging session.

**Supplementary Figure 8**

**Sub-categorization of process-situated microglial Ca^2+^ events observed in Figure 4 pharmacology experiments as localized vs propagative.**

Figure 4 plots the frequency of Ca^2+^ events before and after the application of various drugs. Ca^2+^ events were only classified based on their location, i.e. soma-situated vs. process-situated.

After the application of suramin, clopidogrel and TTX, the soma-situated Ca^2+^ event frequency was dramatically reduced (median < 0.5 event per minute). In contrast, process-situated Ca^2+^ event frequency, whilst noticeably reduced, was not completely abolished.

Thus, process-situated Ca^2+^ events are further classified here based on their spreading propensity, i.e. localized vs. propagative. The change in Ca^2+^event frequency (events/min) before and after drug application are plotted. Each pair of data points represents one microglia cell. Columns indicate cohort means. ****p< 0.0001, ***p< 0.001, **p< 0.01, *p <0.05; Wilcoxon’s matched-pairs sign ranked test, 1-tailed.

**A-B.** Pre- vs Post-ACSF exchange, n = 4 cells. Ca^2+^ events classified as (A) Localized, (B) Propagative.

**C-D.** Pre- vs Post-suramin (300 µM), n = 5 cells. Ca^2+^ events classified as (C) Localized, (D) Propagative.

**E-F.** Pre- vs Post-clopidrogel (30 µM), n= 6 cells. Ca^2+^ events classified as (E) Localized, (F) Propagative.

**G-H.** Pre- vs Post-TTX (3 µM), n = 6 cells. Ca^2+^ events classified as (G) Localized, (H) Propagative.

| **Localized Ca^2+^ event frequency** | | | | | | | | | | |
| --- | --- | --- | --- | --- | --- | --- | --- | --- | --- | --- |
| ***Wilcoxon matched-pairs sign rank test*** | **ACSF** | | | **Suramin (300 µM)** | | **Clopidogrel (30 µM)** | | **TTX (3 µM)** | | |
| **p-value** | 0.3125 | | | 0.0312 | | 0.0469 | | 0.0469 | | |
| **significance** | ns | | | * | | * | | * | | |
| **comparison** | Pre vs Drug | | | | | | | | | |
| **1-tailed or 2-tailed** | 1-tailed | | | | | | | | | |
|  | | | | | | | | | | |
| ***Sample size*** | **Pre** | **ACSF** | | **Pre** | **Suramin** | **Pre** | **Clopidogrel** | **Pre** | | **TTX** |
| **n, cells** | 4 (same as 4C) | | | 5 (same as 4F) | | 6 (same as 4I) | | 6 (same as 4L) | | |
| **outliers removed prior** | not attempted | | | | | | | | | |
| **n, mice** | 3 (same as 4C) | | | 4 (same as 4F) | | 5 (same as 4I) | | 6 (same as 4L) | | |
|  | | | | | | | | | | |
| ***Descriptive statistics*** | ***(events per minute)*** | | | | | | | | | |
|  | **Pre** | | **ACSF** | **Pre** | **Suramin** | **Pre** | **Clopidogrel** | **Pre** | **TTX** | |
| **Minimum** | 7.500 | | 9.400 | 4.500 | 0.1000 | 2.600 | 0.3000 | 1.400 | 1.000 | |
| **25% Percentile** | 9.575 | | 9.475 | 6.500 | 0.2500 | 3.800 | 0.7500 | 4.475 | 1.300 | |
| **Median** | 16.35 | | 10.40 | 13.80 | 1.600 | 4.800 | 2.000 | 8.300 | 2.000 | |
| **75% Percentile** | 17.20 | | 16.73 | 18.40 | 5.350 | 12.03 | 3.025 | 16.28 | 7.050 | |
| **Maximum** | 17.30 | | 18.60 | 19.10 | 6.700 | 12.40 | 4.900 | 22.80 | 14.70 | |
| **Range** | 9.800 | | 9.200 | 14.60 | 6.600 | 9.800 | 4.600 | 21.40 | 13.70 | |
| **Mean** | 14.38 | | 12.20 | 12.72 | 2.560 | 6.783 | 2.083 | 10.07 | 4.267 | |
| **Std. Deviation** | 4.627 | | 4.331 | 6.165 | 2.777 | 4.249 | 1.601 | 7.501 | 5.269 | |
| **Std. Error of Mean** | 2.314 | | 2.165 | 2.757 | 1.242 | 1.735 | 0.6534 | 3.062 | 2.151 | |

| **Propagative Ca^2+^ event frequency** | | | | | | | | | | |
| --- | --- | --- | --- | --- | --- | --- | --- | --- | --- | --- |
| ***Wilcoxon matched-pairs sign rank test*** | **ACSF** | | | **Suramin (300 µM)** | | **Clopidogrel (30 µM)** | | **TTX (3 µM)** | | |
| **p-value** | 0.1875 | | | 0.0312 | | 0.0312 | | 0.0469 | | |
| **significance** | ns | | | * | | * | | * | | |
| **comparison** | Pre vs Drug | | | | | | | | | |
| **1-tailed or 2-tailed** | 1-tailed | | | | | | | | | |
|  | | | | | | | | | | |
| ***Sample size*** | **Pre** | **ACSF** | | **Pre** | **Suramin** | **Pre** | **Clopidogrel** | **Pre** | | **TTX** |
| **n, cells** | 4 (same as 4C) | | | 5 (same as 4F) | | 6 (same as 4I) | | 6 (same as 4L) | | |
| **outliers removed prior** | not attempted | | | | | | | | | |
| **n, mice** | 3 (same as 4C) | | | 4 (same as 4F) | | 5 (same as 4I) | | 6 (same as 4L) | | |
|  | | | | | | | | | | |
| ***Descriptive statistics*** | ***(events per minute)*** | | | | | | | | | |
|  | **Pre** | | **ACSF** | **Pre** | **Suramin** | **Pre** | **Clopidogrel** | **Pre** | **TTX** | |
| **Minimum** | 1.200 | | 0.6000 | 0.1000 | 0.000 | 0.000 | 0.000 | 0.1000 | 0.000 | |
| **25% Percentile** | 1.275 | | 0.6750 | 0.5500 | 0.000 | 0.07500 | 0.000 | 0.1000 | 0.000 | |
| **Median** | 1.900 | | 1.500 | 1.500 | 0.000 | 1.250 | 0.2000 | 0.5000 | 0.1000 | |
| **75% Percentile** | 4.250 | | 3.600 | 2.950 | 0.3500 | 1.900 | 0.5250 | 2.075 | 0.5250 | |
| **Maximum** | 4.900 | | 4.100 | 3.100 | 0.5000 | 2.200 | 0.6000 | 3.200 | 1.200 | |
| **Range** | 3.700 | | 3.500 | 3.000 | 0.5000 | 2.200 | 0.6000 | 3.100 | 1.200 | |
| **Mean** | 2.475 | | 1.925 | 1.700 | 0.1400 | 1.100 | 0.2500 | 1.017 | 0.2833 | |
| **Std. Deviation** | 1.682 | | 1.588 | 1.251 | 0.2191 | 0.9033 | 0.2811 | 1.222 | 0.4665 | |
| **Std. Error of Mean** | 0.8410 | | 0.7941 | 0.5595 | 0.09798 | 0.3688 | 0.1147 | 0.4989 | 0.1905 | |

**Supplementary Figure 9**

**Validation of microglial Ca^2+^ event detection by the AQuA analysis pipeline.**

AQuA analyses raw fluorescence image series by detecting pixels with significant intensity changes over time and clustering them across both spatial and temporal dimensions. These clusters, termed “Ca²⁺ events,” differ from standard ROI analyses in that they can change in area and also translocate between regions, provided the translocation pathway is contiguous.

We validated AQuA against typical ROI-based analysis. Specifically, we compared the ΔF/F traces from AQuA-identified Ca^2+^ events and their equivalent ROIs.

We derived these equivalent ROIs from the total spatial footprint of each AQuA-identified event by cumulatively summing the participating subcellular areas. These areas were contiguous and exhibited Ca²⁺ activity at some point during the event, though not necessarily throughout its entire duration. The average signal within each of these equivalent ROIs was extracted as a raw trace, and the average of the lowest 10% of values was used as F_0_ to compute ΔF/F.

To allow direct comparison of AQuA-derived and ROI-based ΔF/F traces, both were normalized by calculating z-scores as:

**zA = (traceA - mean(traceA)) / std(traceA)**

We then quantified waveform similarity using the Pearson correlation coefficient (PCC) and categorized Ca²⁺ events as 1) soma-situated and localized, 2) soma-situated and propagative, 3) process-situated and localized, 4) process-situated and propagative. The distribution of their PCC scores are plotted as truncated violin plots. In all categories, at least 90% of events showed a PCC score above 0.95, indicating a high degree of similarity and validating our use of AQuA.

|  | **Soma, Localized** | **Soma, Propagative** | **Process, Localized** | **Process, Propagative** |
| --- | --- | --- | --- | --- |
| **n, Ca^2+^ Events** | 79 | 82 | 2249 | 391 |
| **n, cells** | 18 (same as 1D) | | | |
| **n, mice** | 15 (same as 1D) | | | |
|  | | | | |
| ***Pearson correlation coefficient (PCC)*** | | | | |
| **mean** | 0.989990813 | 0.984457808 | 0.990525479 | 0.981242381 |
| **SD** | 0.036619683 | 0.027515582 | 0.05724903 | 0.063366287 |
| **SEM** | 0.004120036 | 0.003038588 | 0.001207184 | 0.003204571 |
|  | | | | |
| ***Proportion of Ca^2+^ events above different PCC thresholds*** | | | | |
| **PCC >0.95** | 96.2% | 91.4% | 98.6% | 93.1% |
| **PCC >0.9** | 98.7% | 95.1% | 99.2% | 98.1% |
